# Tracing the origins of Carnelian ornaments in Northeast Africa: morphological, technological and chemical compositional analyses of beads from medieval and post-medieval upper Nubia, Sudan

**DOI:** 10.1007/s12520-025-02228-0

**Published:** 2025-05-08

**Authors:** Jonathan Mark Kenoyer, Joanna Then-Obłuska, Randall Law, Laure Dussubieux

**Affiliations:** 1 University of Wisconsin, Madison, USA; 2 University of Warsaw, Warsaw, Poland; 3 Field Museum, Chicago, USA

**Keywords:** Nubia, Sudan, Carnelian, Indian ocean trade, Provenance study, LA-ICP-MS

## Abstract

Morphological, technological and chemical compositional analyses of carnelian beads and raw materials from two sites in ancient Sudan are presented. Six carnelian beads are from Banganarti, a Christian pilgrimage center of the Makuria Kingdom (9th -14th centuries) and 18 beads and two carnelian flake fragments are from Old Dongola, capital of the Kingdom of Dongola (14th–18th centuries). Bead shapes show considerable variation, but bead perforations were all done with diamond tipped drills, a technique originating in ancient India and closely associated with South Asian bead workshop traditions. Chemical compositional analyses using laser ablation–inductively coupled plasma–mass spectrometry (LA-ICP-MS) and data processing using both Canonical Discriminate Analysis and Principal Components Analysis, show that many of the beads were made using carnelian that can be sourced to peninsular or western India. Other beads derive from geological sources in Iran, Yemen, Egypt and Türkiye. The two carnelian flakes source to India. These beads provide new insights into issues of technology transfer, stylistic variation and trade networks linking Medieval and post-medieval Nubia to distant regions of West and South Asia.

**Supplementary Information:**

The online version contains supplementary material available at 10.1007/s12520-025-02228-0.

## Introduction

**Fig. 1:**
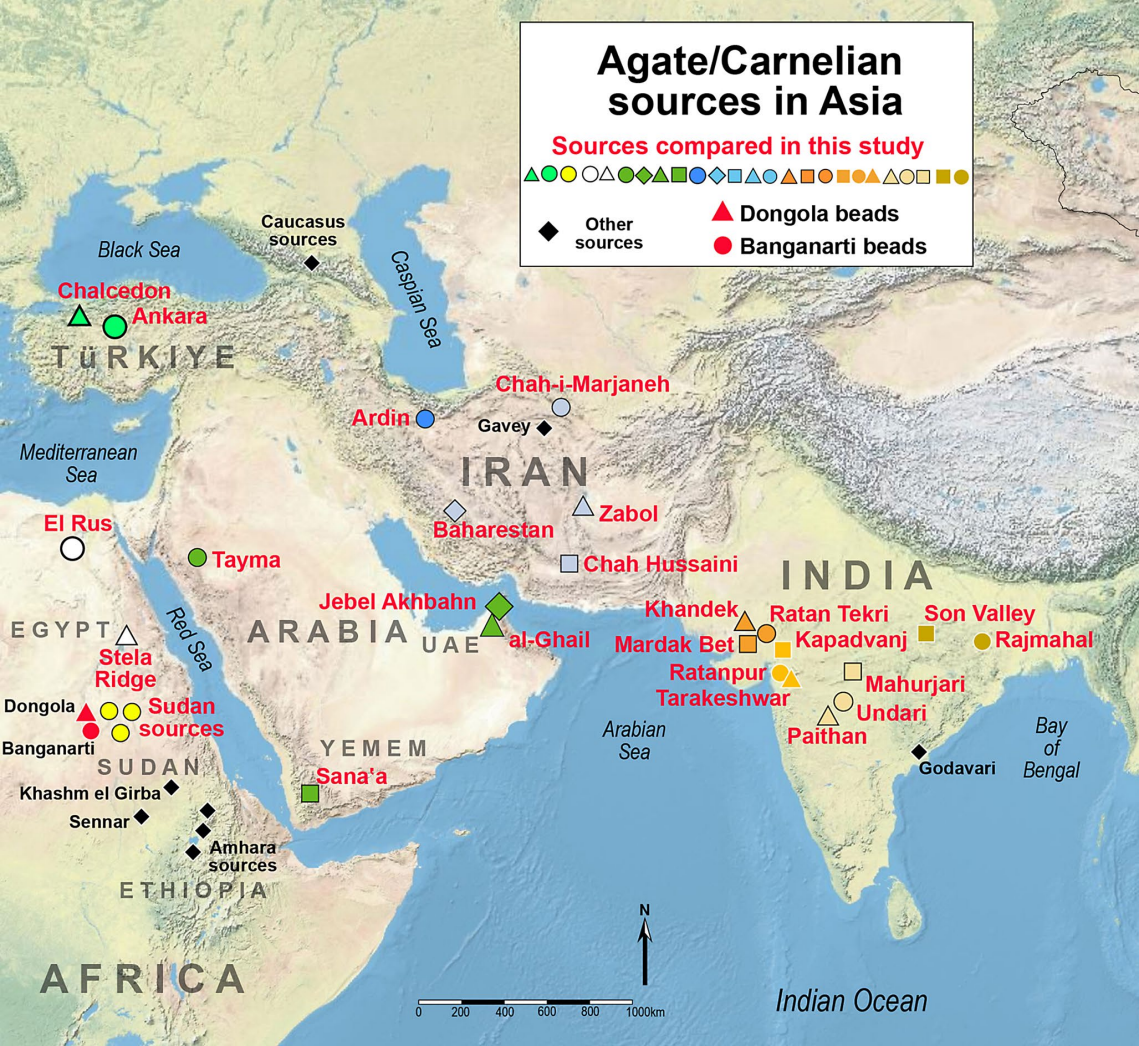
Map of major sites and agate resource areas

The production, use and trade of stone beads has long been of interest to archaeologists and historians because of the many ways in which stone beads intersect with multiple aspects of culture and ideology. One of the most important types of stone bead used by ancient and historical communities in South Asia, West Asia and northeast Africa is the red-orange form of chalcedony, commonly referred to as carnelian. This paper presents the results of morphological, technological and chemical compositional analyses of carnelian beads and raw materials from two sites in ancient Sudan. Six carnelian beads derive from the 9th -14th century layers of Banganarti (18°10’05.9"N 30°46’21.0"E), a Christian pilgrim site active during the Makuria Kingdom (Then-Obłuska et al. 2021; Żurawski 2006; Drzewiecki 2017) and 18 beads and a two flake fragments dating to between the 15th–18th centuries come from excavations at Old Dongola (18°13’23.5"N 30°44’38.0"E), a capital of the Kingdom of Dongola (Then-Obłuska 2022; Obłuski and Dzierzbicka ) (Fig. 1).

**Fig. 2:**
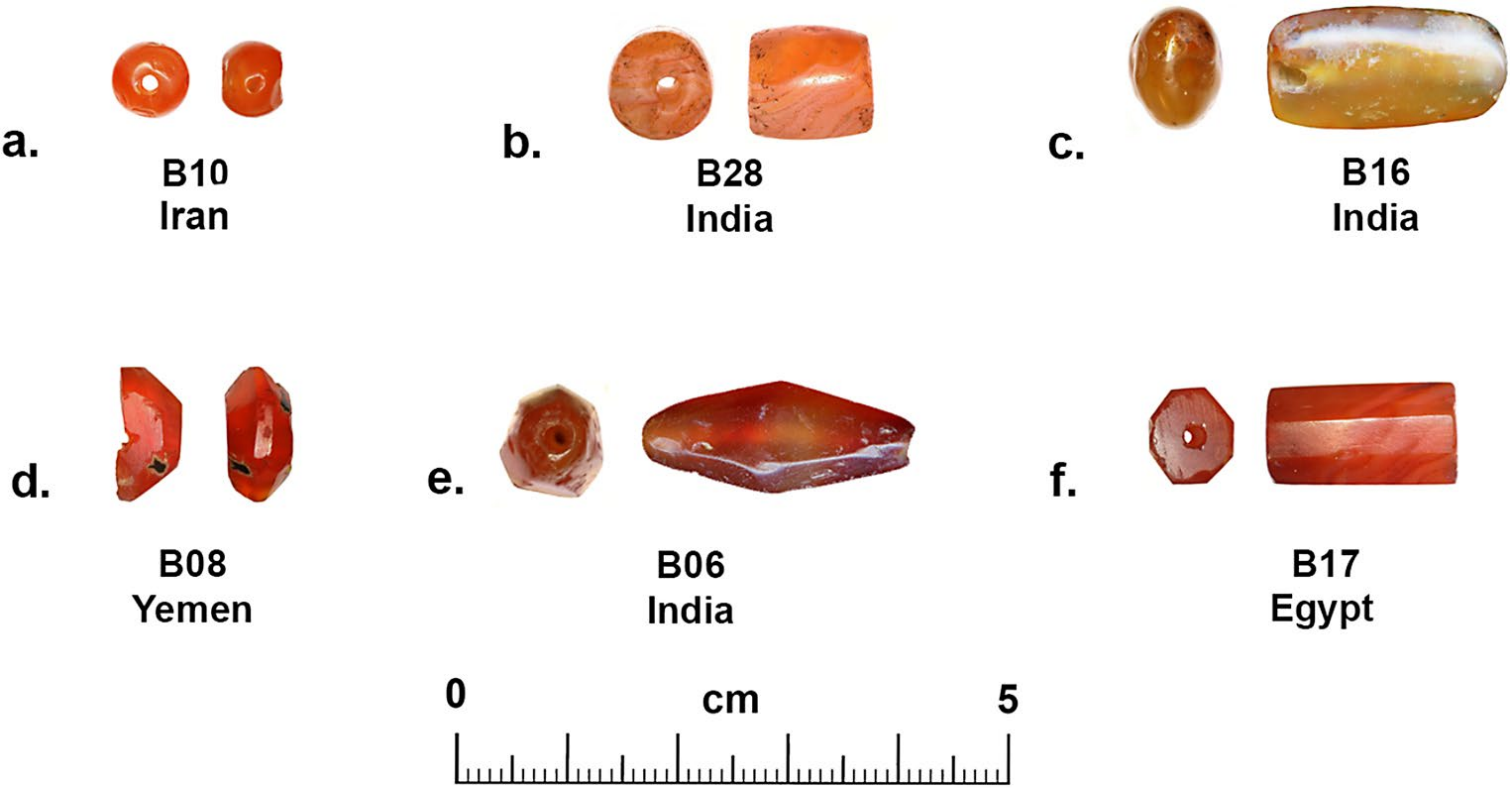
Banganarti carnelian beads (Raw material, cross section, longitudinal section, number, source). **a**. Carnelian, circular, short barrel, B10, Iran. **b**. Carnelian, circular, long barrel, B28, India. **c**. Carnelian, oval, long irregular barrel, B16, India. **d**. Carnelian, octagonal? faceted (broken), short octagonal, B08, Yemen. **e**. Carnelian, heptagonal faceted, long bicone, B06, India. **f**. Carnelian, octagonal faceted, long tapered octagonal, B17, Egypt

**Fig. 3:**
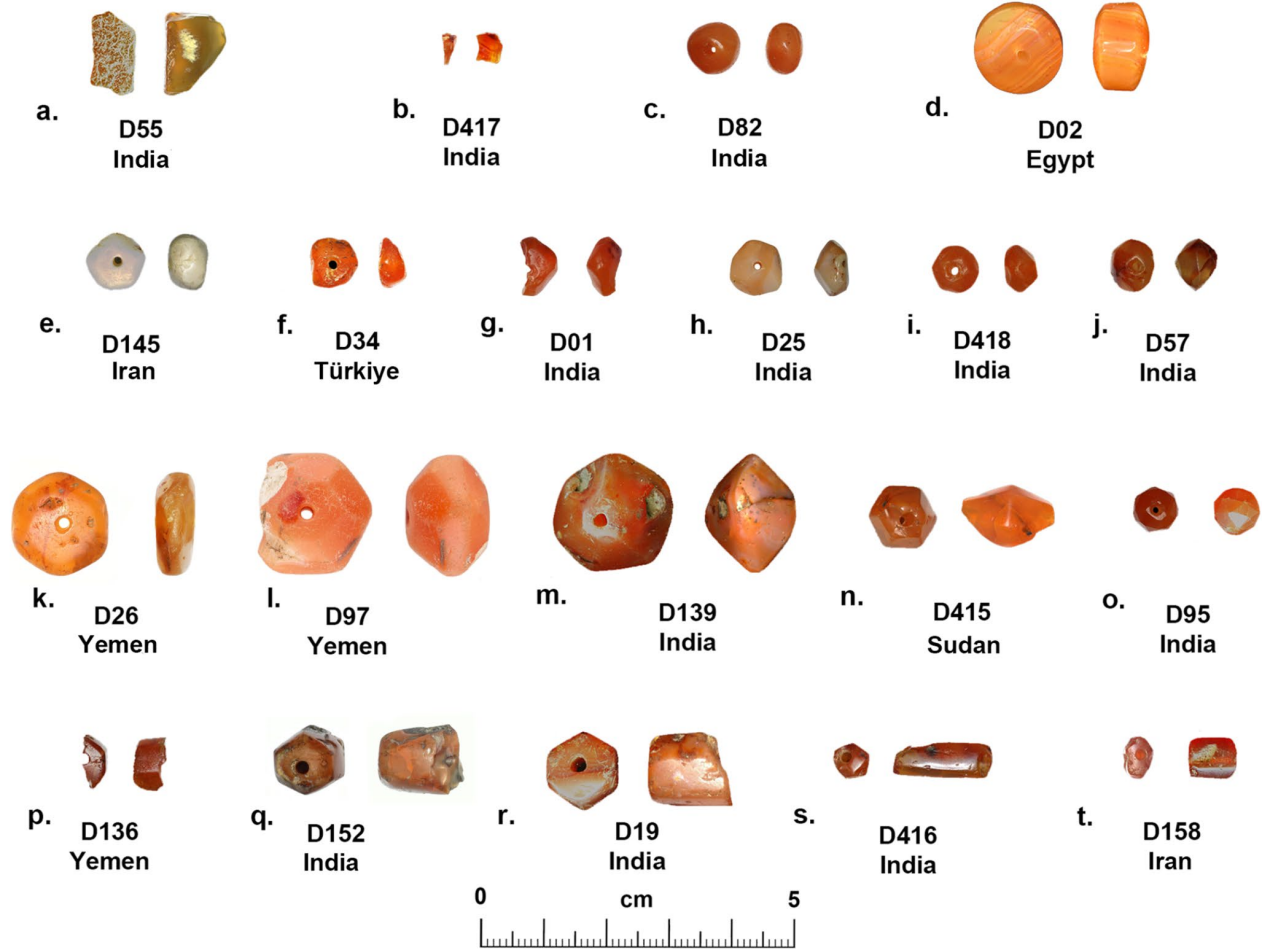
Dongola carnelian flakes and beads (Raw material, cross section, longitudinal section, number, source). **a**. Carnelian flake fragment with cortex, yellow orange, partly heat treated, rolled, D55, India. **b**. Carnelian flake fragment, red-orange, heat treated, D417, India. **c**. Carnelian, irregular circular, short oval, D82, India. **d**. Carnelian, circular, short rectangular with convex ends (short cylindrical), D02, Egypt. **e**. Carnelian, burned and whitened, irregular pentagonal faceted, short irregular hexagonal, D145, Iran. **f**. Carnelian, irregular pentagonal (broken), short irregular pentagonal, D34, Türkiye. **g**. Carnelian, hexagonal/octagonal? faceted (broken), short irregular hexagonal, D01, India. **h**. Carnelian, hexagonal faceted, short irregular hexagonal, D25, India. **i**. Carnelian, hexagonal faceted, short irregular octagonal, D418, India. **j**. Carnelian, hexagonal faceted, short irregular hexagonal, D57, India. **k**. Carnelian, hexagonal faceted, short octagonal, D26, Yemen. **l**. Carnelian, with cortex, hexagonal faceted, short octagonal (broken), D97, Yemen. **m**. Carnelian, with cortex, heptagonal faceted, short irregular octagonal, D139, India. **n**. Carnelian, hexagonal faceted, long bicone (broken), D415, Sudan. **o**. Carnelian, heptagonal faceted, short decagonal (44 facets), D95, India. **p**. Carnelian, hexagonal? faceted, long rectangular (broken), D136, Yemen. **q**. Carnelian, hexagonal faceted, long rectangular (broken), D152, India. **r**. Carnelian, hexagonal faceted, long rectangular (broken), D19, India. **s**. Carnelian, hexagonal faceted, long rectangular (broken), D416, India. **t**. Carnelian, irregular octagonal faceted, irregular long tapered octagonal, D158, Iran

Various qualities of carnelian as a raw material are found in widely dispersed and diverse geological deposits in from South Asia to north Africa, but the size and quality of the raw materials, and the technologies used to produce beads from this hard rock developed along different trajectories in each region. During the prehistoric and early historical periods, shaping and perforation of beads in Egypt (Nai 2014 (1932); Gorelick and Gwinnett 1983), Mesopotamia (Gwinnett and Gorelick 1987; Moorey 1994), Arabia (Hausleiter; Hausleiter et al. 2019; Purschwitz 2017), Iran (Lume Pereira et al. 2017; Piperno 1973) and South Asia (Kenoyer 2017a) was carried out using various forms of chipping, grinding, sawing, pecking and drilling with either stone or abrasive drills (Kenoyer 2003). However, around 600 BCE, the development of diamond tipped drilling technology in South Asia, radically transformed the organization and scale of carnelian bead production (Kenoyer 2017a). Morphological studies of the beads from Banganarti (Fig. 2) and Old Dongola (Fig. 3) show considerable variation in shape and size, which could indicate major changes in bead shapes over time as well as the fact that they may originate from very different workshop traditions (Ludvik et al. 2022). The quality of the manufacturing is also highly varied, with some beads showing traces of multiple stages of production (e.g. chipping, coarse grinding, fine grinding, etc.) that were not removed before the bead was finally polished, while others are exquisitely made with careful faceting and polish. However, the bead perforations were all done with double diamond tipped drills, a technique originating in ancient India and closely associated with South Asian workshop traditions (Kenoyer 2017a). There are, however, some variations in the sizes of drills used and the ways in which the beads were drilled that could indicate different workshops or regional styles of production within South Asia or in regions where diamond drilling technology spread. The study of stone bead production stages, chipping, grinding, polishing and particularly the perforation or drilling of beads, provides a unique insight into the development and change in technology within a given society and can also help to understand the spread of technology when unique aspects of production can be traced across space and time.

The sourcing of rocks used to make stone beads is also a valuable tool for archaeologists to reconstruct economic networks and long-distance exchange. Stone beads are often reused and passed down from one generation to the next, resulting in long histories of use, often stretching over millennia (Ludvik et al. 2022:115). The immense amount of information that can be gleaned from multifaceted studies of stone beads has increased significantly in the past several decades due to the application of various high-resolution scientific techniques. Among the most important techniques are those using chemical characterization for geologically sourcing specific types of stone or rock raw materials. The most efficient and minimally destructive technique for analyzing the chemical composition of a rock is the use of Laser Ablation-Inductively Coupled Plasma-Mass Spectrometry (LA-ICP-MS) (Law et al. 2013; Carter and Dussubieux 2016; Kenoyer et al. 2022). There are however significant challenges when using chemical sourcing techniques, the most critical being the development of a robust comparative database that has examples from all potential sources of raw materials, in this case carnelian or agate. Another challenge is to sample sufficient examples of rocks from a single geological formation to capture the full variability of its composition. The current database used for this study has more than 50 discrete sources for carnelian and between 10 and 20 samples per source area (see below for more discussion of the sampling and analysis).

Compositional analysis using laser ablation–inductively coupled plasma–mass spectrometry (LA-ICP-MS) and data processing using both Canonical Discriminate Analysis (CDA) and Principal Components Analysis (PCA), show that many of the beads from Banganarti and Dongola were made using carnelian that can be sourced to peninsular or western India. The shapes of the beads from Indian sources are comparable to beads of similar date found in South Asia as well as in sites in East Africa (Kenoyer et al. 2024). Other beads include shapes that are similar to those produced in South Asia, but they appear to have been made of carnelian from geological sources in Yemen, Egypt, Iran and Türkiye. Two carnelian flakes from Old Dongola source to India, but it is not clear if they represent local production of beads since no carnelian bead workshop has been discovered at the site. Finally, there are a few bead shapes that may reflect local styles, but the perforations were made with diamond drills that reflect a technology from South Asia. The use of diamond drilling technology for the production of carnelian beads from different source areas and having a diverse morphology requires a new interpretive framework to investigate the possibility of drilling technology transfer to regions outside of South Asia, or the presence of workshops with Indian trained craftspeople in widely dispersed regions. There are also some important stylistic similarities across several different regions that need to be considered, as well as regional variation in some shapes. Finally, it is critical to reexamine the nature of trade networks linking Medieval and post-medieval Nubia to distant regions of West and South Asia.

## Background

The discussion and study of the carnelian bead trade between ancient India or South Asia and the West, particularly the Red Sea and northeastern Africa has a long history, beginning with the early accounts in the Periplus and later classical Roman authors (Schoff 1912; Casson 1989). Various types of agate and carnelian from ancient India were important trade items and were in high demand in the Roman world and have been reported at the Roman port of Berenike on the Red Sea (Then-Obłuska 2018). Carnelian continued to be exported through the Red Sea during Aksumite rule and there is evidence for carnelian beads at the Aksumite Red Sea port of Adulis and what appear to be Indian style diamond drilled beads at the capital city of Aksum, Ethiopia itself dating to around the 5th century (Phillipson 2000; Harlow 2000: 83–86, Fig. 65b). Other evidence for early trade is seen at the Nubian Red Sea port of Suakin, where large quantities of imported pottery, porcelain, and glass from Yemen, the Persian Gulf region, India, China, and Southeast Asia attest to Nubia’s strong eastern connections (Phillips 2019:459–460). Further inland, North Indian glass beads have been confirmed at the medieval and post-medieval Nubian sites of Banganarti, Dongola, and Soba (Then-Obłuska and Dussubieux 2022, 2025 In pressThen-Obłuska et al. 2021; Then-Obłuska and Dussubieux 2023a, b).

Later European colonial accounts highlight the importance of the port city of Cambay or Khambhat (Fulljames 1838/1839; Summers 1851; Arkell 1936) as well as some important details of trade between western India, Arabia and the regions of northeastern Africa, including Sudan (Arkell 1945, 1946). Accounts by Arkell of excavations of burials near the palace site of Uri in Darfur, western Sudan, report the presence of Indian agate and carnelian beads dating to around the 16th century (Arkell 1936; Plate 3, Fig. 22; Arkell 1946: 193, 196, Plate XVIa). The most recent study of carnelian trade between ancient India and East Africa focused on the coastal regions of Tanzania and Kenya, where there is clear evidence for the import of a range of different types of carnelian beads dating to between the 7th and 15th centuries (Kenoyer et al. 2024). Some of the shapes found in these coastal regions are identical to those found in Banganarti and Old Dongola, particularly the short spherical beads, the oval or circular barrel shapes and various faceted shapes. The presence of similar shapes of Indian carnelian beads in both coastal East Africa and inland Sudan and Ethiopia, suggests that traders were carrying these beads along multiple routes to diverse markets and that this trade was carried on for several hundreds of years.

Somewhat later in time, the Italian traveler Giovanni-Battista Brocchi, who was in Sennar, the capital city of the Funj Kingdom of Sudan between 1825 and 1826 (Fig. 1), noted the presence of both agates and carnelian. Among the luxury goods that were brought by caravans from the port of Suakin on the Red Sea, he states that the most prized, and perhaps also the most expensive were the banded chalcedony or agate ornaments (*rubanė*). He also notes that carnelian, called *akek*, were not considered very valuable and were equivalent to coral beads (Brocchi 1843:472–473). It is not clear from this account why carnelian was not considered to be valuable, but the fact that it was part of long-distance trade commodities indicates that there was probably no local production of carnelian beads during this time period. The lack of a robust local carnelian industry in Sudan at this time is surprising given the extensive carnelian industry in Egypt to the north during earlier Pharaonic times (Nicholson and Shaw ) and the fact that carnelian nodules are available in alluvial deposits and desert areas of Sudan itself (Aston et al. 2000:26–27; Harrell 2010:73; Then-Obłuska 2013:118). Independent of their market value at the beginning of the 19th century, carnelian ornaments have been found in archaeological excavations of medieval and post-medieval sites in Nubia (Then-Obłuska et al. 2021) and this study builds on the work of earlier scholars by focusing on beads excavated in the heartland of Sudan along routes that linked East Africa and the Red Sea ports to the upper Nile River system as well as regions of western Sudan (Fig. 1).

### Banganarti site and Carnelian beads

By the early eighth century, Christian Nobadia in Lower Nubia (regions of the First and Second Nile Cataracts) and Makuria in Upper Nubia (regions of the Third and Fourth Cataracts) were united under the Makurian king. Throughout the Makurian period (late 4th -13th century), numerous churches and monasteries were built in Nubia (Obłuski 2019). Banganarti is located on the right bank of the Nile, some 10 km upriver of the Makurian capital at Old Dongola (Fig. 1). The churches excavated at Banganarti include a large church accompanied by smaller buildings and an enclosure wall (Żurawski 2012, 2014; Żurawski ). The role of Banganarti as an important pilgrimage center is demonstrated by the large number of inscriptions in Greek, Old Nubian and other languages that have been found on walls of buildings throughout the site, apparently left by pilgrims from as far as southern France and Yemen (Łajtar 2020; Łajtar and Płóciennik 2011). The evidence for long distance interactions is further corroborated through the study of glass beads that can be traced to Iranian, Egyptian and Levantine production centers from the same time period (Then-Obłuska and Dussubieux 2022). However, the source for carnelian beads and their potential import from South Asia or possibly from other regions had never been studied in depth until the current study.

Banganarti is a mound covering two superimposed churches, subsequently labelled the Lower and Upper Churches, located in the sandy area separating the village from the riverbank fields (Żurawski 2004; Żurawski 2021). The Lower Church reveals a painted wall decoration with figures of saints accompanied by Greek inscriptions (Żurawski 2004). After the Lower Church walls had been levelled, probably in the mid- 10th century, another church, the Upper Church, was constructed. A row of seven chapels was constructed above the sepulchers, and their apse walls were adorned with painted portraits of Nubian kings and high dignitaries. A huge building was added to the church’s western wall, the ‘Western Building’, otherwise called the ‘Residential Building’ (Żurawski 2004, 2014). Ongoing fieldwork has also revealed a large settlement complex within the defensive walls, which functioned from the late sixth or early seventh century to the 16th century or later (Drzewiecki 2008, 2017; Żurawski et al. 2017). More than 2,150 beads and pendants were found in excavations at Banganarti between 2006 and 2020 and they were made of organic and inorganic materials (resin, ostrich eggshell, stone, faience, glass) with glass beads dominating the assemblages. Among all these beads, only 10 were made of carnelian and of these a total of six samples were analyzed using LA-ICP-MS (to be discussed below) (Fig. 2). Each of the six carnelian beads have a different shape and reflect different technologies and production processes or *chaîne opératoire* (Kenoyer 2017a; Roux 2000): circular short barrel, circular long barrel, oval long irregular barrel, octagonal faceted short octagonal, heptagonal faceted long bicone and octagonal faceted long tapered octagonal (Fig. 3a to f respectively). Detailed descriptions of each bead, including measurements, contexts and dating are provided in SI Appendix 1.

### Dongola site and Carnelian beads

Around CE 1500, new Arab powers arose in eastern Sudan around Suakin and indigenous tribes in the south, near the White Nile. In the latter case, in the early 16th century a group known as the Funj founded the great Funj Dynasty at Sennar, which expanded to incorporate most of the Nilotic and eastern Sudan. The Third Cataract became a frontier between the Ottoman Empire in Egypt and the Funj Empire in Sudan from the late 16th century until 1821 (Fisher 2012:42). The Kingdom of Dongola became the border zone between the Ottoman and the Funj territories, and the capital city remained an important political and trade center until the end of the Funj period (El-Bushra 1971; O’Fahey and Spaulding 1974:25–26; Obłuski and Dzierzbicka 2021). The recent excavations at Old Dongola by the Urban Metamorphosis of a Medieval African Capital City (UMMA) European Research Council project has revealed extensive evidence for trade contacts during the period between the 14th–18th century. For example, a few thousand glass beads that were studied can be linked mainly to European production regions and only some of them were of East Mediterranean, Middle Eastern or South Asian origin (Then-Obłuska and Dussubieux 2022, 2025 In press). The sources of the carnelian beads however were not known although it has long been assumed that they probably originated in ancient India or South Asia.

In 2018 the UMMA (Urban Metamorphosis of a Medieval African City) Project started the systematic excavations of the Old Dongola capital (Obłuski and Dzierzbicka; Obłuski et al. 2021)(Fig. 1). The works concentrated mainly in sector one that encompassed the citadel. Apart from the specimens found on the surface or in the rubbish fill of the first layers, the excavated beads of diverse materials (ostrich eggshell, marine mollusk shell, stone, faience, ceramic, glass) were located inside and outside the city walls, and even on top of the city wall and can be generally dated to between the 15th and 18th century. A total of 18 carnelian beads and three flake fragments were recovered (Fig. 3), and of these all but one flake were studied and analyzed using LA-ICP-MS. Three of the beads have a circular cross section (Fig. 3b, c, d) and various longitudinal sections. All the other beads are faceted shapes (Fig. 3e to t), with 5, 6, 7 and 8 facets in the cross section and 4, 6, 8 and 10 facets in the longitudinal section. The range of faceted bead shapes from Dongola include types that were commonly made in workshops in western India (e.g. Limbodara and Khambhat) for export to Islamic markets in Arabia and eventually were traded down the line to Africa and beyond (Kenoyer et al. 2024). Detailed descriptions of each bead, including measurements, contexts and dating are provided in SI Appendix 2.

As will be discussed in more detail below, the combined morphological, technological and chemical analyses of carnelian beads from these two Upper Nubian sites, medieval Banganarti and post-medieval Dongola, show that the inland regions of northeastern Africa were connected to multiple distant resource areas between the 9th and 18th centuries. This complements and refines our knowledge based on the evidence from glass beads and other historical accounts (Then-Obłuska and Dussubieux 2022; Then-Obłuska et al. 2021).

### Stone bead Documentation

The documentation of the stone beads from the two sites includes detailed measurements of the exterior bead shape as well as the manufacturing indicators visible on the bead surface and ends, as well as the shape of the drill hole itself (Kenoyer 2017b) (SI Fig. 1). A digital caliper was used for the basic measurements and the drill hole shape was recorded by making a mold of the drill hole using dental impression material (3 M Express Regular Set Light Body) (Kenoyer 2017c). The beads were photographed using both a digital camera and a digital microscope to gain high resolution images of the bead surfaces. A digital microscope was used to take images of the drill hole impressions at 20x and 50x in order to calculate depth of drilling and drill hole taper value (Ludvik et al. 2022). The drill hole taper value is calculated by measuring the width of the drill hole where it starts at one end of the bead (W1) and the width at the end of the drilling (W2) as well as the distance between the two points (D1). The drill hole taper value (DHTV) is calculated with the formula (W1-W2/D1 = DHTV). This measurement provides additional confirmation regarding the type of drill used in perforation and idiosyncratic aspects of drilling.

### Bead shape terminology and illustration

**Fig. 4:**
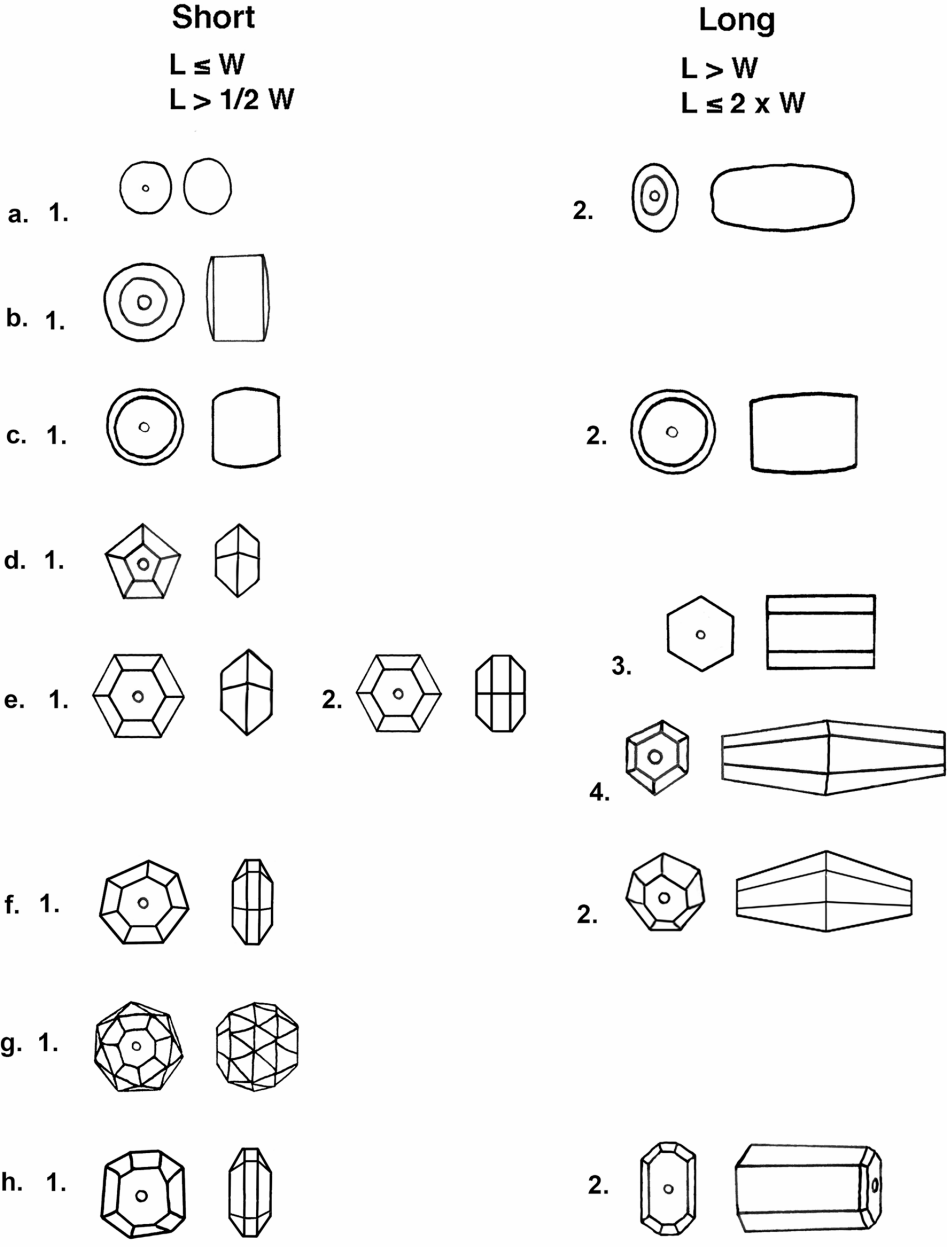
Banganarti and Dongola Bead Types. **a**. 1. irregular circular, short oval, 2. oval, long irregular barrel. **b**. 1. circular, short rectangular with convex ends (short cylindrical). **c**. (1) circular, short barrel, (2) circular, long barrel. **d**. 1. pentagonal faceted, short hexagonal. **e**. (1) hexagonal faceted, short hexagonal, (2) hexagonal faceted, short octagonal, (3) hexagonal faceted, long rectangular, (4) hexagonal faceted, long biconical. **f**. (1) heptagonal faceted, short octagonal, (2) heptagonal faceted, long bicone. **g**. 1. heptagonal faceted, short decagonal (44 facets), **h**. (1) octagonal faceted, short octagonal, (2) octagonal faceted, long tapered octagonal

The terminology used to describe the beads and the illustration format is based on the early models provided by Horace Beck (Beck 1928), with minor variations developed in subsequent studies of South Asian beads (Fig. 4Kenoyer 2017b). For bilaterally symmetrical beads, first the geometrical shape of the cross section is defined, e.g. circular, oval, hexagonal faceted, etc. Then the longitudinal section is defined in combination with maximum diameter-to-length ratios to determine, short to very long bead shapes (Kenoyer 2017b). Due to the awkward wording for terms such as bi-truncated oval or bi-truncated bicone, the common terms of “barrel” or “bicone” are used for easy reference. For each site, the archaeological contexts and dating as well as the bead identification, raw material, and measurements are presented in SI Appendices 1 and 2. A summary of the major manufacturing processes represented in these two assemblages is provided below before discussing specific beads from each site.

### Carnelian bead production and drilling

**Fig. 5:**
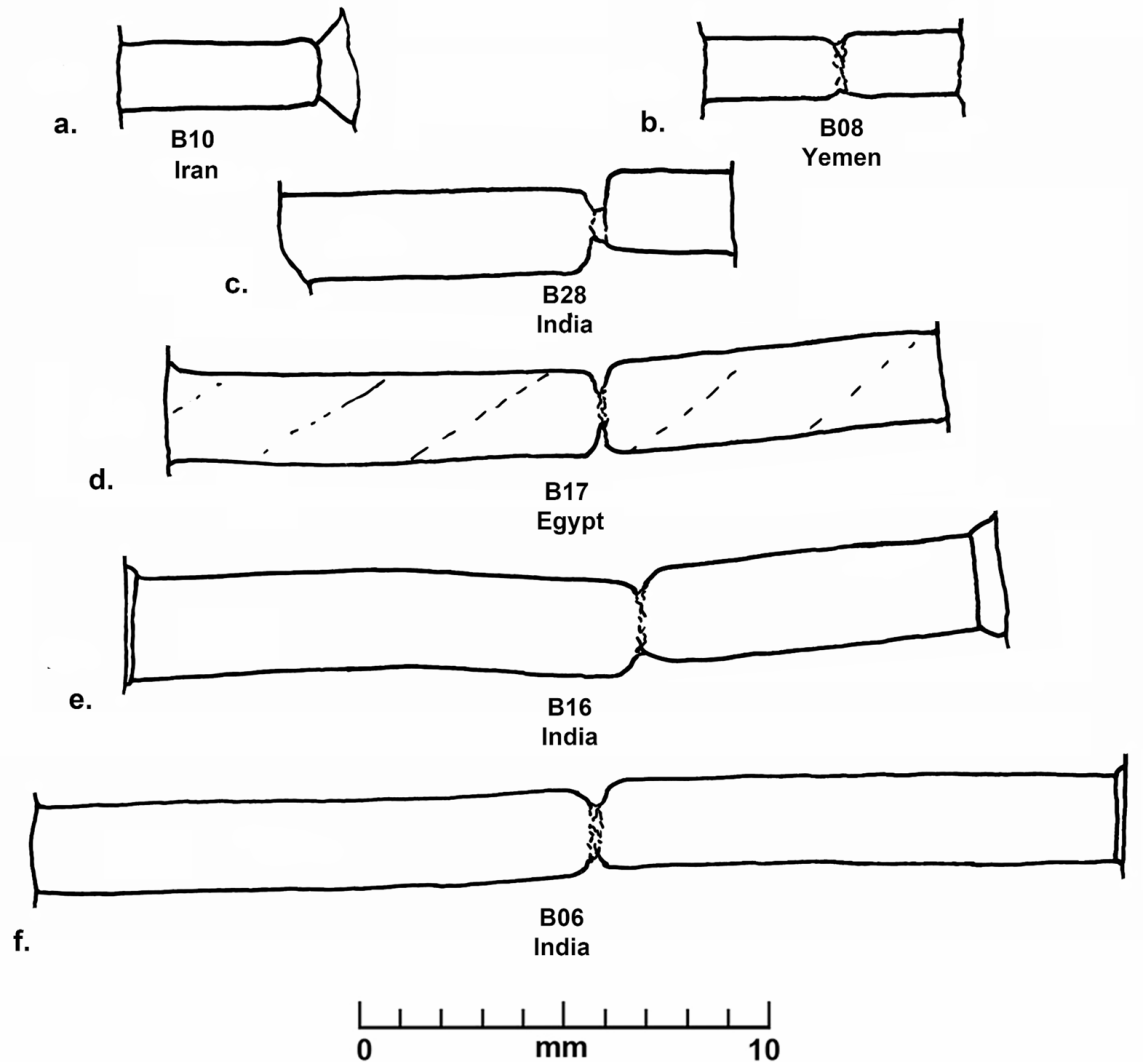
Banganarti Drill hole profiles. **a**. Straight cylindrical, drilled from one side and popped out, B10, Iran. **b**. Straight cylindrical, drilled from two sides, centered, approximately equal drilling, B08, Yemen. **c**. Straight cylindrical, drilled from two sides, not centered, one side longer, B28, India. **d**. Straight cylindrical spiraling, drilled from two sides, centered, one side longer, B17, Egypt. **e**. Stepped straight cylindrical, drilled from two sides, centered, one side longer, B16, India. **f**. Straight and stepped cylindrical, drilled from two sides, centered, one side longer, B06, India

The carnelian beads from both sites were produced from relatively good quality carnelian that was probably originally collected as rolled nodules (7 to 10 cm in length) that were then broken down into smaller blocklets after initial heat treatments. This process is well documented at the sites of Khambhat and Nagra, Gujarat, where production has been going on since the before the 3rd century BCE, with substantial increase during the Medieval (CE 500 to 1500) and Colonial Periods (CE 1500–1900) up to the present (Kenoyer 2024; Trivedi 1964; Roux 2000; Bhan et al. 2017). After preliminary heat treatment, bead roughouts and finer bead blanks are prepared using the inverse indirect percussion technique with an iron stake and buffalo horn hammer that is still practiced in Khambhat today (Kenoyer et al. 1994). Many of the beads from both Banganarti and Dongola still show traces of flake scars from the initial shaping that were not removed by subsequent grinding. A few beads still show evidence for the original cortex of the nodule itself. Beads that still retain the flake scars or cortex reflect more expedient production with less time devoted to grinding and polishing, resulting in beads that have irregular or flawed surfaces. The quality of production reflected in these beads indicates that the consumers were mainly concerned with a specific shape and size and possibly color of the bead and that minor imperfections were ignored. Most of the faceted beads have relatively irregular sizes of facets, that would have been made by hand on a flat grinding stone or rotary lapidary wheel, either horizontal or perpendicular. There is no indication for precise faceting that results from special devices used to hold the beads at specific angles during the faceting process. Final polishing of the faceted beads would also have been carried out on similar turning devices, with the use of fine abrasives (emery or brick dust) embedded in lac (insect resin) or applied to wooden lapidary wheels. Although most of the beads appear to have been polished individually, one spherical bead from Banganarti (B10, Fig. 5) has possible evidence for the use of mass polishing in a leather bag with an abrasive slurry. This technique was most commonly used on spherical or barrel beads (Kenoyer et al. 1991:54).

The carnelian beads from both sites were perforated using diamond tipped drills that included the use of single diamond starter drills followed by double diamond drills for the deeper perforations. This perforation process is distinctive of the drilling technology that developed in ancient India as early as 600 BCE and was widespread in South Asia by around 300 BCE (Kenoyer 2017a). Generally speaking drilling with double diamond drills results in a straight cylindrical hole that has a mean drill hole taper value (DHTV) of 0.00 mm with a range from − 0.01 to 0.01 mm (Ludvik et al. 2022; Table 1). The mean DHTV based on the measurement of 42 drill holes in the combined Banganarti and Dongola beads is also 0.00 mm, but the range is from − 0.05 to 0.02 mm. This pattern indicates that the bead drilling included some drill holes that were slightly wider at the tip of the hole due to drilling angles or redrilling. The drill hole taper values for each bead are presented in SI Appendices 1 and 2.

**Fig. 6:**
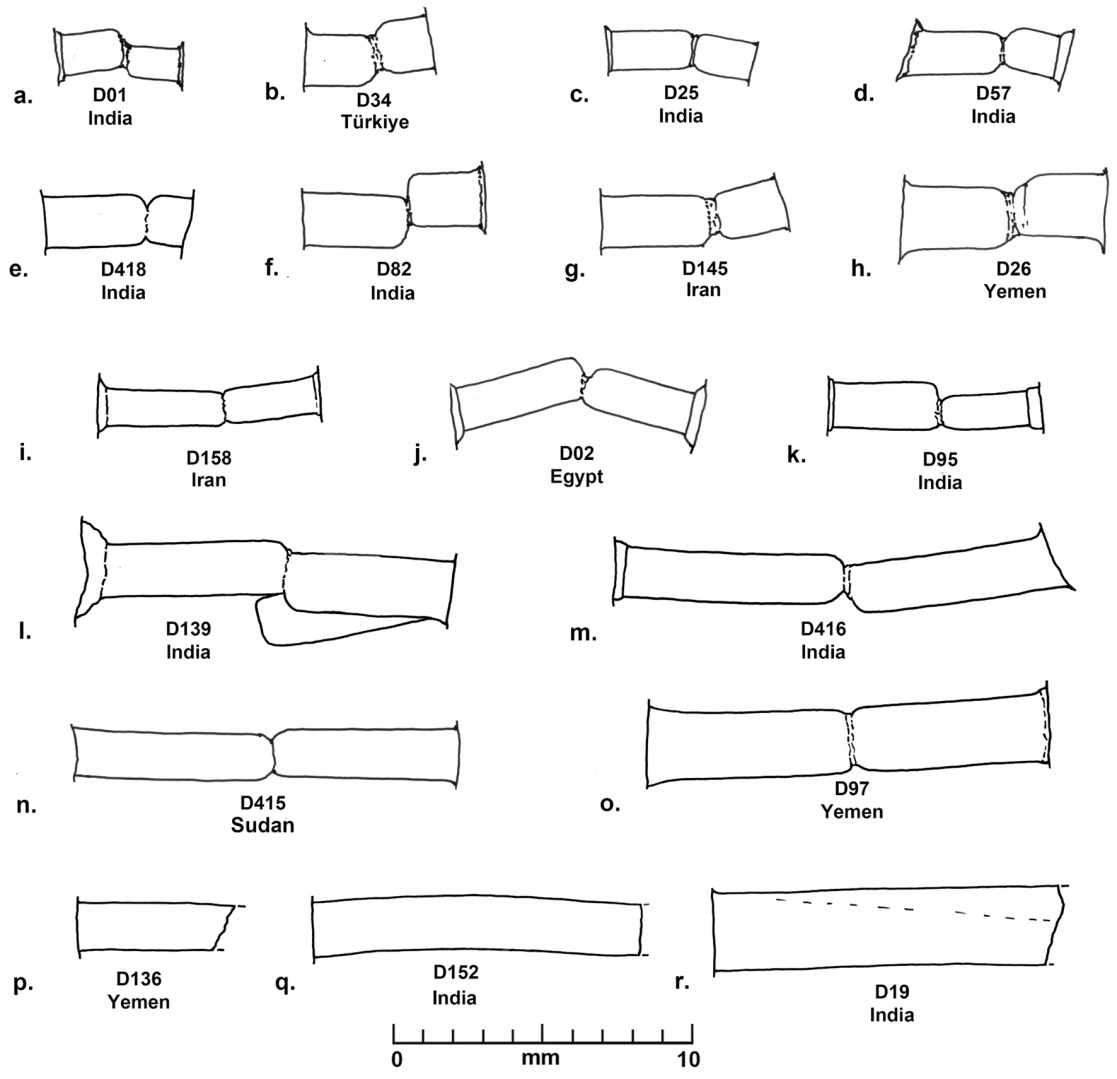
Dongola Drill hole profiles. **a**. Stepped straight cylindrical, drilled from two sides, one drill thinner, not centered, one side longer, D01, India. **b**. Straight cylindrical, drilled from two sides, not centered, one side longer, D34, Türkiye. **c**. Stepped and straight cylindrical, drilled from two sides, not centered, one side longer, D25, India. **d**. Stepped cylindrical, drilled from two sides, not centered, one side longer, D57, India. **e**. Straight cylindrical, drilled from two sides, one drill thinner, centered, one side longer, D418, India. **f**. Straight and stepped cylindrical, drilled from two sides, not centered, one side longer, D82, India. **g**. Straight cylindrical, drilled from two sides, one drill thinner, centered, one side longer, D145, Iran. **h**. Straight cylindrical, drilled from two sides, centered, one side longer, D26, Yemen. **i**. Stepped cylindrical, drilled from two sides, centered, one side longer, D158, Iran. **j**. Stepped cylindrical, drilled from two sides, angled, not centered, one side longer, D02, Egypt. **k**. Stepped cylindrical, drilled from two sides, one drill thinner, centered, one side longer D95, India. **l**. Stepped and straight cylindrical, drilled from two sides, double angled drilling, not centered, one side longer D139, India. **m**. Stepped and straight cylindrical, drilled from two sides, angled, not centered, one side longer D416, India. **n**. Straight cylindrical, drilled from two sides, centered, approximately equal drilling, D415, Sudan. **o**. Straight cylindrical, drilled from two sides, centered, approximately equal drilling, D97, Yemen. **p**. Straight cylindrical drill hole, broken bead, D136, Yemen. **q**. Straight cylindrical drill hole, broken bead, D152, India. **r**. Straight cylindrical slightly spiraling drill hole, broken bead, D19, India

The silicone impressions of the drill holes also make it possible to compare the depths of drilling from each side, the angle of drilling and how centered the two drill holes are (Figs. 5 and 6). To facilitate drilling with a double diamond tipped drill the bead driller usually uses a drill with a single large diamond chip on the tip to create a depression that is often still visible after the drilling is completed (Figs. 5e and 6a, c, d, f, i, j, k, l, m and o). The use of a starter drill makes it easier to position the double diamond drill at the center of the bead end. If the double diamond drill tip is wider than the depression formed with the single diamond drill, then this starter depression is obliterated during the drilling process. Some beads were drilled only from one side and the opposite end was popped out with the pressure of the drilling, leaving a large negative flake scar on one side (Fig. 5a). In most other examples the beads were drilled from two sides with the perforations meeting somewhere in the middle or center of the bead. When the drilling was done precisely with equal depth of drilling from both sides, it indicates that the bead driller had carefully calculated the distance needed for drilling from one side and then after repositioning the bead in the vise it was drilled in a precise perpendicular alignment to meet the drilling from the first side (Figs. 5b and 6n and o). When drill holes are centered it is easy for the thread to be strung through the bead and reduces the potential for leaving sharp edges at the tip of the perforation inside the bead that can abrade or cut the string. When a bead driller is not very skilled or does not take the time to center the drills the edge of the drill hole is slightly off center or angled, resulting in a much smaller hole for threading the beads and also resulting in a sharp edge inside the middle of the bead that can cut the string (Figs. 5c, and 6j, k). When drilling from the second side, the bead driller must be careful to stop drilling before actually reaching the opposite drill hole as it is possible to dislodge one of the tiny diamond bits at the end of the drill if the drill gets stuck in the opposite hole. When the drilling from the second side gets near to the other hole it usually results in a tiny flake popping through the hole. Careful examination of the internal flake scars using the silicone impression and the SEM makes it possible to determine which side of the drilling was done first and which was done second (Figs. 6k and 12c). Usually, the drilling from the first side is slightly longer than that from the second side. Sometimes the same drill or same size of drill was used for drilling from both sides, while in other instances the bead driller used a different size of drill that was either thicker or thinner than the first drill. The diversity of drilling patterns seen in the drilling distance and centering of the drill holes can be used to define patterns of individual bead drillers or workshop traditions (Ludvik et al. 2022). Although the sample of beads studied from the two sites is quite small, they do show a wide diversity of drilling patterns, and this suggests that the beads were made in many different workshops by multiple drillers (Figs. 5 and 6).

### Banganarti beads

**Fig. 7:**
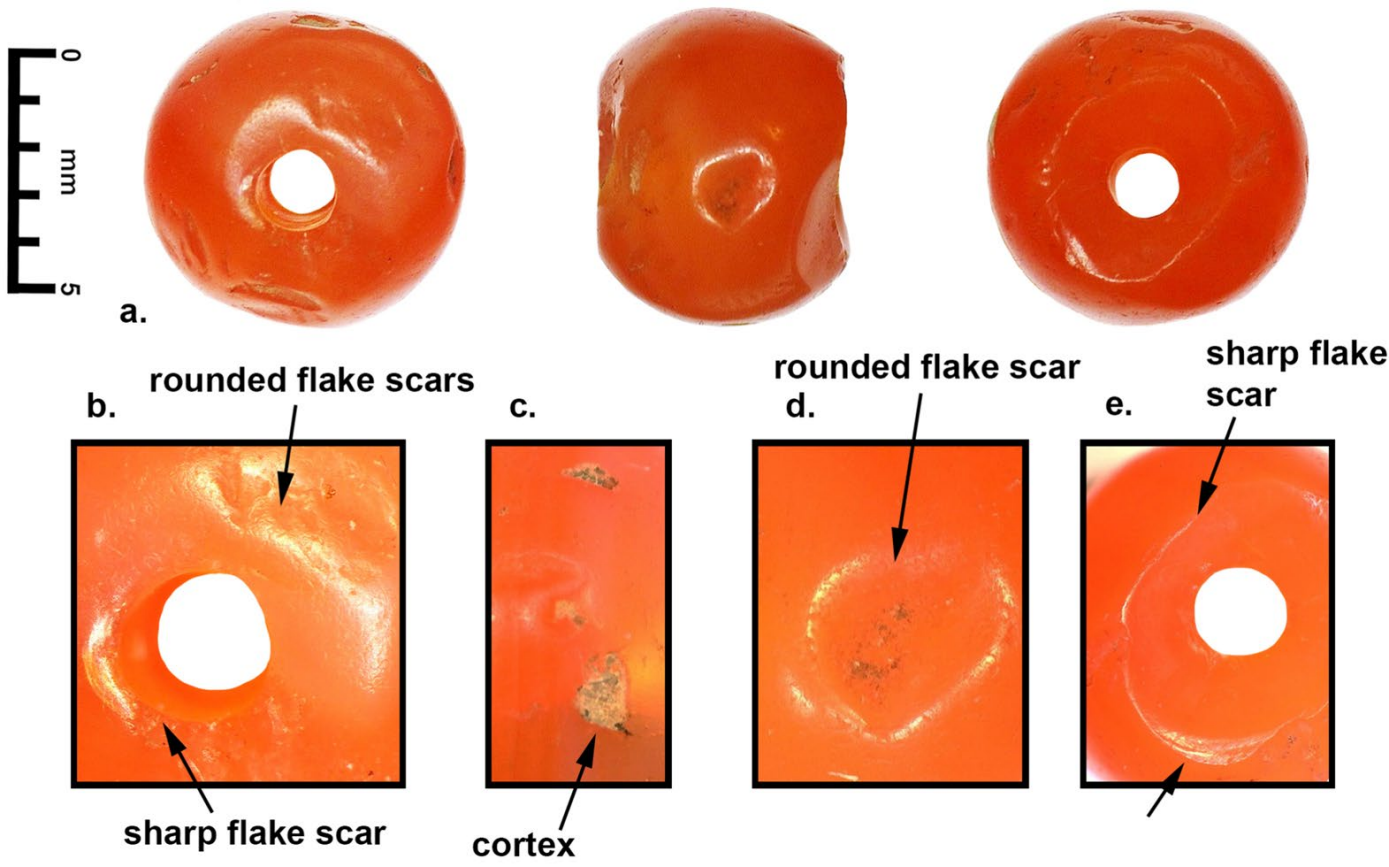
**a**. Carnelian, circular, short barrel, B10, Iran, **b**. rounded flake scars from bag polish and sharp flake scar from drilling after polishing, **c**. trace of cortex, **d**. rounded flake scars from bag polish, **e**. sharp flake scar from drilling and popping out after polishing

**Fig. 8:**
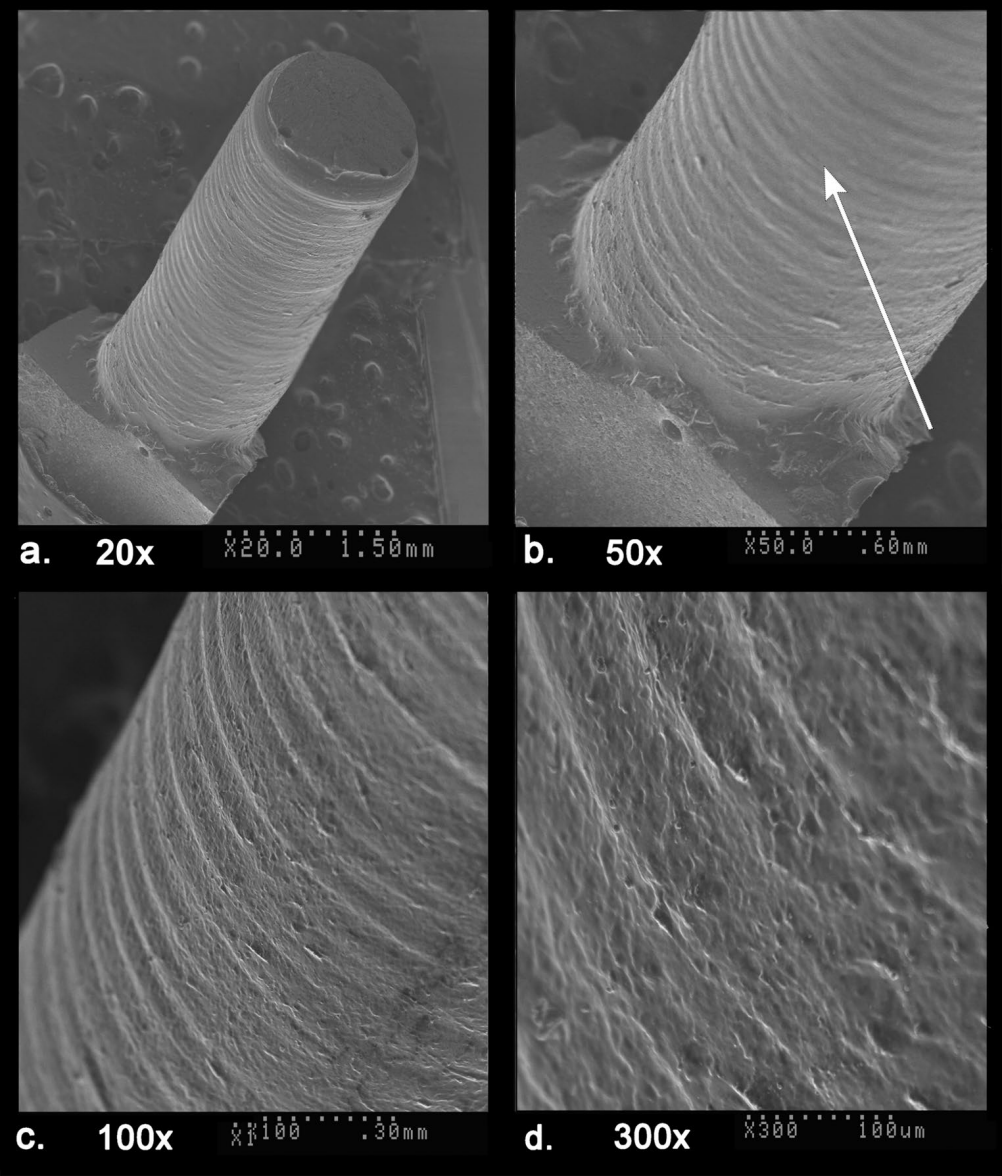
SEM of bead B10 drill hole; **a**. tilted view showing parallel grooves, 20x, **b**. smooth area from string wear, 50x, **c**. 100x, **d**. 300x

A short description of the six carnelian beads from Banganarti documented in this study were originally reported in a preliminary site report (Then-Obłuska et al. 2021), and here they will be discussed in more detail with additional SEM analysis and updated information on their sourcing (Fig. 2). One bead (B10) is a short spherical bead drilled from one side and popped out with traces of original nodule cortex and flaking on the exterior and low luster polish that could be the result of bag polish prior to drilling (Fig. 7a). The flake scar on the exterior has its edges rounded in a way that could indicate mass polishing in a bag (Fig. 7d), but the drilling was done after the polishing as the flake scar from the drilling has a relatively sharp edge (Fig. 7b and e). This type of bead is typical of expedient bead production where beads are positioned in a vise and drilled only from one side. The lack of proper finishing indicates that the consumers were not concerned about minor flaws in the bead surface or the large negative flake scar that distorts the spherical shape of the bead. The SEM analysis of the bead drill hole shows clear parallel striae of a double diamond drill with some string wear at the edges of the drill hole (Fig. 8). In the earlier analysis this bead was sourced to India (Then-Obłuska et al. 2021), but with our updated geochemical database including carnelian from some additional source areas it is now sourced to Iran. As will be discussed below in more detail, the carnelian may still originate from India but has some chemical anomalies that put it closer to some Iran sources. However, it is also possible that the bead was made in Iran or Afghanistan using local carnelian but was produced by Indian craftspeople or local craftspeople who were using the same technologies practiced in the workshops in historical India (this would include sites in what is now Pakistan). Another possible explanation is that Iranian carnelian raw material was imported to a workshop in South Asia and the bead was produced in South Asia by local craftspeople. Evidence for the import of Iranian carnelian to South Asia has been documented in the ongoing studies of carnelian beads and raw materials from sites in the Indus Valley Tradition, dating from the Early Harappan and Harappan Periods (2800 − 1900 BCE) (ongoing studies by Kenoyer and Law).

**Fig. 9:**
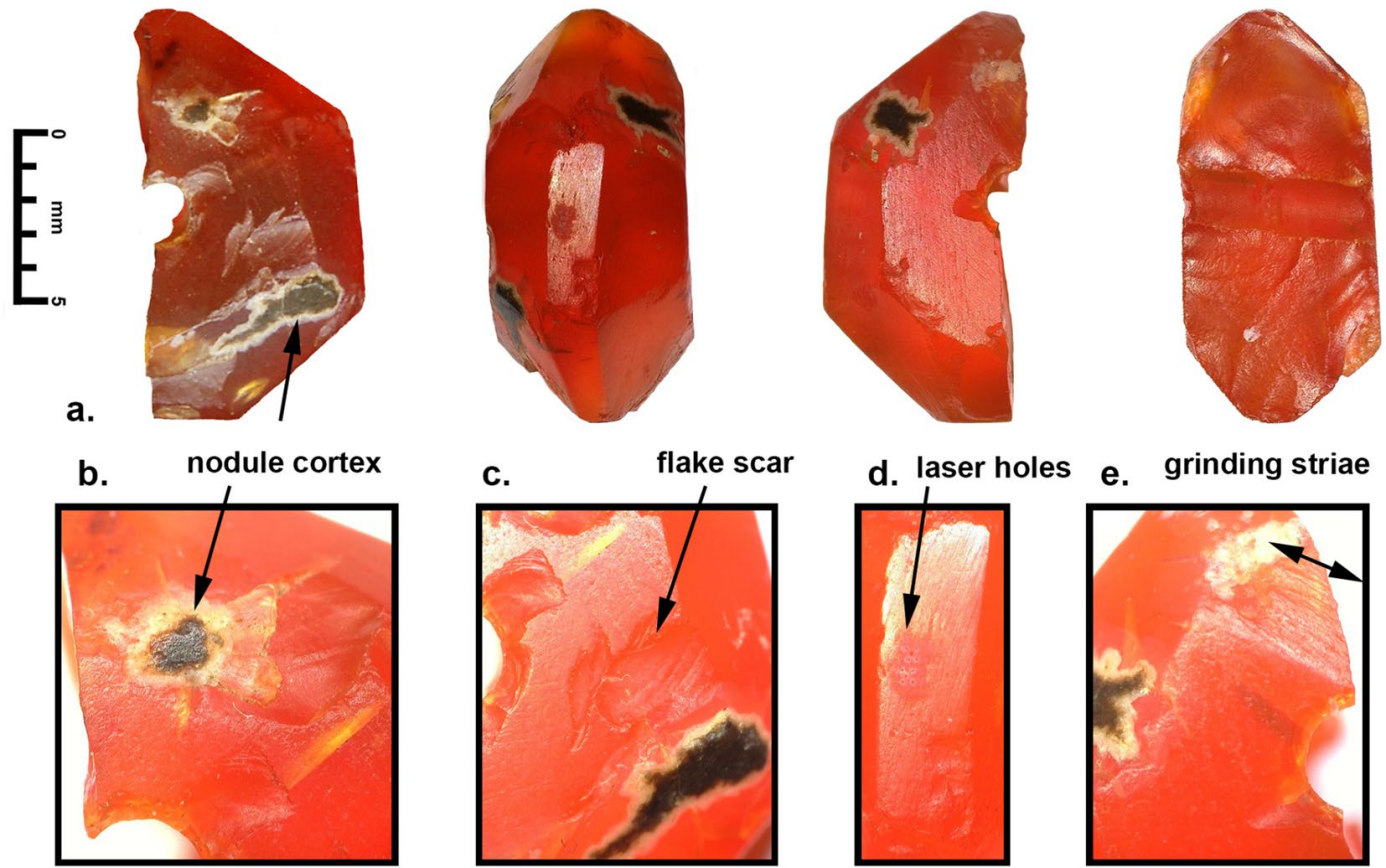
**a**. Carnelian, octagonal? faceted (broken), short octagonal, B08, Yemen, **b**. nodule cortex remnant, **c**. flake scar from initial chipping, **d**. laser ablation holes, **e**. faceted surface with rough grinding striae still visible

A faceted bead with hexagonal cross section and short octagonal longitudinal section sources to Yemen (B08) (Fig. 9). This bead fragment was split longitudinally and shows drilling from both sides with a double diamond drill. The external surface was only partly ground and polished leaving several original flake scars and patches of nodule cortex (Fig. 9). The fact that the nodule cortex is found on both sides of the bead indicate it was made from a relatively small nodule. In the agate mines of western India, most nodules are relatively large and measure between 5 and 7 cm in length and 3 to 5 cm wide. Very small agate nodules are not usually collected for preparing beads as they require too much labor to remove all the cortex and expose the carnelian. The drilling technology involves the use of double diamond drills from both sides and the drill sizes are relatively similar and the drilling is well centered. The external surfaces are not faceted symmetrically, and the traces of rough flake scars, cortex and rough grinding traces indicate a lower quality of production than is seen in many beads produced in South Asian workshops. It is possible that this bead was made in a workshop somewhere in Yemen, but the precise location is unknown. This bead shows heavy wear on the facets and also some string hole wear that indicates it was used for a long time before it was broken and discarded.

Historically, Yemen is well known for the deep red quality of its carnelian, and there are some references to the many different types of agate and carnelian products crafted in the workshops of Sana during the period around 1290–1295, including various sizes of small and large rosaries or prayer beads (Jasem 1999:3). However, it is not known if the bead perforations were made using diamond drilling technology or an earlier technology involving metal drills and abrasives. An early study of carnelian beads from the site of Hajar-ar-Rayhani in Yemen had incorrectly identified the use of diamond drilling in beads dated to the 3rd century BCE (Gwinnett and Gorelick 1991:193), but subsequent studies by the Kenoyer and Geoffrey Ludvik have shown that this and other similar beads found at the site were actually drilled using metal and fine abrasives (work in progress) (Ludvik et al. 2022; Fig. 8).

Another faceted bead (B17) is octagonal in cross section and slightly tapered rectangular in longitudinal section (SI Fig. 2). Although in an earlier publication this bead was thought to source to India (Then-Obłuska et al. 2021), with our current database the carnelian for this bead now sources to Egypt. The perforation was done with a double diamond drill, and the overall shape and form are very similar to beads produced in South Asia. The drilling is well centered, and the SEM image shows that there is a slightly spiraling feature in the drill hole form (SI Fig. 3a), a pattern that is often seen in beads produced in South Asian workshops. The remaining three beads from the site all source to India and show the use of double diamond drilling technology. Two of the beads are relatively common shapes; long barrel (B28) (SI Fig. 4) and long heptagonal faceted bicone (B06) (Fig. 2e). The third bead (B16) has an oval cross section and a long irregular barrel longitudinal section that is not typical of beads produced in the Indian workshops (Fig. 2c).

### Dongola flakes and beads

Two of the carnelian flake fragments from Dongola that were analyzed both source to India. One of the flake fragments (D55, Fig. 3a) has the original nodule cortex on the dorsal face of the flake and the edges are rounded from abrasion that is typical of colluvial gravels. The color of the flake is a dull yellow grey that indicates it is probably unheated carnelian. The other flake fragment (D417, Fig. 3b) is semi translucent with a deep red orange color that is typical of heated carnelian. The edges of the flake fragment are relatively sharp, and it may represent manufacturing debris or a fragment of a broken bead, but there is no trace of any original bead surface preserved on the fragment. Normally when carnelian bead manufacture is present at a site, the flakes from chipping and broken bead blanks are found throughout the excavations, but the fact that only three total flakes (two studied here) were found in the excavations suggests that these objects are not indicative of local bead production. Future excavations may be able to provide new information on this issue.

The carnelian beads from Dongola are mainly faceted shapes except for two beads that have simple forms. One of these beads (D82) has an irregular circular cross section and a short oval longitudinal section and the carnelian sources to India (Fig. 3c). The bead was perforated from two sides with a combination of single and double diamond drills of approximately the same size, but the alignment of the drilling was off center (Fig. 6f). The other bead (D02) sources to Egypt and has a circular cross section and a short rectangular longitudinal section with slightly convex ends (Fig. 3d). This bead was drilled with single and double diamond drill from two sides (Fig. 6j), but the drilling angles are not aligned perpendicularly and barely meet to allow for a completed perforation. This suggests that the bead driller was not that skilled, since the bead is relatively short, and it should not have been that difficult to align the drilling. The SEM image of the drill hole impression shows fine parallel grooves from rotary double diamond drilling (SI Fig. 5). All the remaining faceted beads were drilled from two sides, using combinations of single and double diamond drilling technology, but the sizes and shapes of the drills are quite varied. One bead (D145) that sources to Iran (Fig. 3e) has a perforation with single and double diamond drilling from two sides with very clear parallel grooves from rotary motion seen in the SEM image (SI Fig. 6). However, another bead sourced to Iran (D158) (Fig. 3t) shows a slightly thinner drill and surface features that are less distinct in terms of horizontal rotary grooves visible in the SEM image (SI Fig. 7). Two beads sourcing to Türkiye (D34) and Yemen (D26) show very similar sizes of double diamond drilling in their SEM images though the bead shapes are quite different (Fig. 3f and k). All but one of the faceted beads show relatively irregular faceting to create the final form with significant variation in the quality of production and finishing (see SI Appendix 2 for details). The fact that similar faceted bead shapes are made from carnelian that source to India, Iran, Türkiye, Yemen, and Sudan suggest that the use of diamond drilling technology and broadly similar workshop production styles spread to many regions connected through Islamic trade networks. However, the quality of production and drilling does not appear to have been strictly regulated.

**Fig. 10:**
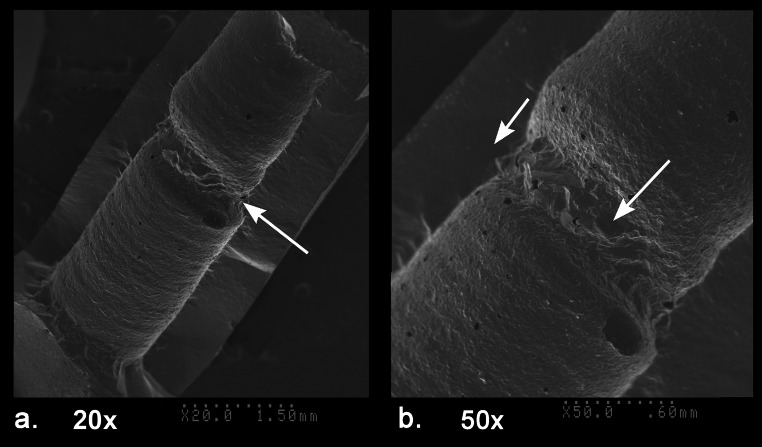
SEM of bead B08 drill hole; **a**. tilted view showing centered drilling from two sides and traces of fractured surface connecting the two drill holes, 20x, **b**. detailed view of flake scar patterns that indicate the pressure for the fracture comes from the shorter drilling side

**Fig. 11:**
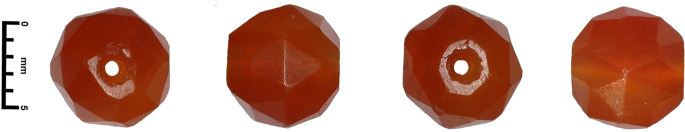
**a**. Carnelian, heptagonal faceted, short decagonal (44 facets), D95, India. Bead ends show chipping around the edges of the drill hole

**Fig. 12:**
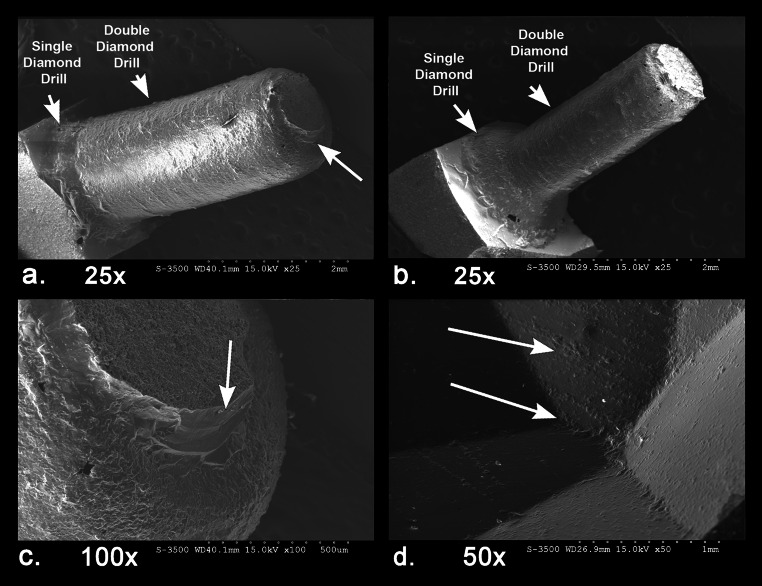
SEM of bead D95 drill hole; **a**. tilted view showing single and double diamond drilling from side one with a relatively thicker drill, faint parallel grooves, and flake scar where the second drilling fractured through, 25x, **b**, tilted view showing single and double diamond drilling from side two with a much thinner drill, faint parallel grooves, 25x, **c**. detail of the tip of the side one drill hole showing the direction of the flake scar resulting from side two drilling, **d**. exterior faceted surface showing rough parallel grinding striae and battered edges of the facets that result from use wear or post depositional abrasion

In contrast to the irregular faceting of most beads from the site, there is one exquisitely faceted bead (D95) (Fig. 10 and 11) with 44 facets that sources to India and provides an example of the high quality of crafting that was undertaken in some Indian workshops. The SEM analysis of the drilling shows it was drilled with a single and double diamond drill perforated from two sides (Fig. 12a-c). One interesting feature of this bead perforation is that it was drilled with a larger drill from one side first, and then a thinner drill from the second side that just barely met with the other drill hole (Fig. 12c). The percussion flake that popped through shows that the pressure was directed from the side of the thinner drill, indicating that this was the second drilling. The edges of the drill hole show traces of string wear that indicate the bead was worn on a cord, possibly as a rosary or a necklace. Finally, the faceted external surfaces of the bead show the degree of precision that was possible with handheld faceting tools (Fig. 12d). The fact that the facets do not meet precisely indicates that they were made using only visual positioning and not with any mechanical device as is now commonly used when faceting stones. Some of the facets still show traces of both the coarse and fine abrasives used in shaping and polishing, and the facet edges show slight rounding from use wear. It is possible that this bead was used in a high-quality rosary or other form of ornament that resulted in external surface wear.

### Sourcing Carnelian bead using LA-ICP-MS

The chemical composition of the beads from Banganarti and Dongola were assessed using Laser Ablation - Inductively Coupled Plasma - Mass Spectrometry (LA-ICP-MS) to determine the probable geologic source (or sources) of the raw material used to produce them. The results from this analysis were compared to an extensive database of potential sources from across African and Eurasia. Although not uniform in appearance, all of the stone used for making the beads from the two sites can be classified as *carnelian* – a translucent to semi-translucent, reddish-orange variety of *agate*, which is itself a member of a broader family of highly variable ornamental microcrystalline silicates that includes (but is not limited to) chalcedony, chrysoprase, sard and onyx (Butler 1995). While the precise geologic mechanisms behind their formation has been the subject of considerable debate (Pabian and Zarins 1994; Moxon 1996; Götze et al. 2001), it is known that “the majority of agate deposits worldwide are related to SiO_2_-poor (andesites, basalts) and SiO2-rich (rhyolites, rhyodacites) volcanic rocks” (Götze 2020:2). The reddish-orange appearance of the variety known as carnelian is due to the presence of elemental iron (Fe). This iron constituent can either be acquired during its initial formation or infused in it later if the stone, after eroding from its original parent-rock, is reconsolidated within iron-rich, lateritic sediments. An iron-enriched agate turns red when heated as the iron within the stone is thermally oxidized. This heating may be natural, caused either by geothermal processes or when an agate is exposed on the surface and subjected to the heat of the sun over a long period of time. However, the highest quality carnelian (i.e., material that is largely free of fractures) is almost always a human-made product. Unweathered, iron-rich agates that have not been naturally heated tend to have a greyish yellow to light brown appearance. The painstaking process of turning agates of this kind into carnelian by subjecting them to multiple, carefully controlled heating has been documented at the major bead-making center of Khambhat, Gujarat by numerous scholars (Janaki 1980; Kenoyer et al. 1991, 1994; Roux 2000) and will not be reviewed here.

Over the past decade, Kenoyer, Law and Dussubieux have been working in collaboration with numerous other researchers to obtain and analyze geologic samples from regionally representative of agate-carnelian deposits (Fig. 1). Over 50 individual sources spanning an area that extends from East Africa to East Asia, are currently represented in this database. Although the preliminary comparisons of the Dongola and Banganarti results to agate sources were made using the entire database (which includes sources in regions as distant as Mongolia and Japan), the results we present here focus on the most pertinent regions, namely, India, Iran, Arabia, Türkiye and northeast Africa. Admittedly, the database is markedly deficient in terms of geologic sources within Africa. Currently, the continent is represented by two discrete geological sources in Egypt (Stele Ridge and El Rus) and a single “source” designated “Sudan” that is actually made up of geologic samples collected from multiple localities in the northern part of the country (Bayuda desert, Has el Guruf and the Meroe area). Regarding carnelian occurrences in Sudan, Then-Obłuska has noted (Then-Obłuska 2013) other carnelian sources further south at Khashm el Girba and in the bed of the Blue Nile in the Sennar-Kasala region. If the result for the beads from Dongola and Banganarti are to be fully understood, it will be necessary to collect and analyze additional samples from northern Sudan as well as from potential sources in other areas of the country, in adjacent nations such as Ethiopia, and, eventually, from more distant regions of the continent. In this way, we might best differentiate local agate/carnelian sources from one another and distinguish local stone from that originating in regions further afield. It is important to note, however, that much of the agate/carnelian of Sudan and Egypt likely originated in Oligocene epoch (31–26 Ma old) volcanic formations that “were originally joined but later became separated by the development of the Red Sea rift” (Petránek 2004: 197). Thus, we might eventually discover that agate sources found across large parts of northeastern Africa and southwestern Arabia have broad geochemical similarities. We have observed such similarities among agate samples from the 10 deposits we have analyzed within the extensive volcanic formation of India known as the Deccan Traps. In that region, it is not always possible to definitively state that an agate artifact came from a precise deposit, only that it appears to have been made from Deccan agate. However, this level of resolution is usually more than sufficient when examining types of materials that are often traded over extremely long distances. When agate sources in Iran, Arabia and Türkiye are also brought into consideration, a full picture of carnelian exchange networks spanning an area from India to Eastern Africa is possible.

The use of LA-ICP-MS to geochemically characterize agate-carnelian beads and sources has previously been employed to determine the geologic provenience of agate-carnelian beads from Southeast Asia (Carter and Dussubieux 2016), Mongolia (Kenoyer et al. 2022) and, most recently, from the Kenyan and Tanzanian sites of Manda, Unguja Ukuu and Kwa Mgogo (Kenoyer et al. 2024). This technique provides highly accurate and precise data on both the major and minor oxide and trace element composition of solid materials. Most importantly, however, it is minimally destructive to artifacts under examination. Using a minute but powerful laser, agate/carnelian beads are sampled at multiple points and the material vaporized by the laser is carried in helium gas to the adjacent ICP-MS where its elemental composition is measured. While the laser does leave behind a series of 60 μm (0.006 mm) diameter pits (Fig. 9d), they are almost invisible to the naked eye. Geologic samples and artifacts were analyzed under the direction of Dr. Laure Dussubieux at the Field Museum of Natural History in Chicago, USA, using a Thermo ICAP Q Inductively Coupled Plasma - Mass Spectrometer that is connected to an ESI-Elemental Scientific Laser - NW213. Full details related to the instrumental and analytical parameters of this study, as well as a large-portion of the agate-carnelian source database itself, will be published shortly (Kenoyer et al. *in press* 2025). The data for the 55 major and minor elements measured for the carnelian beads from Dongola and Banganarti are listed in SI Appendix 3.

Following LA-ICP-MS analysis, initial comparisons of the beads from Dongola and Banganarti were made to our geologic source database using several methods including bivariate plotting of individual elements (or ratios of two elements) against one another, hierarchical cluster analysis and principal component analysis (PCA). However, because most samples making up the database are from known, secure contexts it was decided that primary data evaluation and artifact assignment was best undertaken using *canonical discriminant analysis* (CDA). CDA is a statistical method that uses multivariate data specifically to maximize separation (discrimination) between defined groups (Baxter 2015:185–214) that are in this case, sets of geologic samples collected from individual agate/carnelian deposits. Artifacts (agate/carnelian beads) are treated as unknowns (ungrouped cases) and are assigned a *predicted group membership* (PGM) based on the group’s center (centroid) to which they are nearest in multi-dimensional space. The 1st PGM is that group to which an unknown is nearest (most closely resembles) while the 2nd PGM is the next nearest. Having information on the 2nd PGM is often helpful when making interpretations of individual beads, especially compositional outliers. One important aspect to note is that CDA assumes that a dataset being evaluated contains all possible groups. Thus, an unknown case (artifact) will be assigned a PGM regardless of whether or not the group (source) to which it actually belongs is present in the database. As noted above, there are many potential agate-carnelian sources in northeast Africa and beyond that have not yet been sampled and analyzed. The PGMs to be discussed below should be only considered preliminary determinations of a bead’s geologic provenience until a larger and more comprehensive database for agate sources in Africa itself has been assembled.

Using CDA, the beads from Dongola and Banganarti were compared to 25 agate/carnelian sources in six regions – India (11 sources), Iran (5), Arabia (4), Türkiye (2), Egypt (2) and Sudan (1). The 1st and 2nd PGMs resulting from that comparison are listed in SI Appendix 4 PGM, Section A. Of the six Banganarti beads analyzed, three (B06, C16, B28) have both a 1st and 2nd PGM in one of the Indian sources. Having artifacts assigned both a 1st and 2nd PGM in geologically related sources within the same region strengthens an interpretation that the raw material from which they were made is indeed from that region. The remaining three beads from Banganarti have 1st and 2nd PGMs that are regionally mixed. One bead – B17 – has a 1st PGM in an Indian source with the 2nd PGM in Egypt. Although the 1st PGM in an India source can be taken at face value, the 2nd PGM might indicate that this particular bead is a composition outlier of the Egyptian source that was misidentified. Likewise, bead B10, which has a 1st PGM in one of the Iranian sources, might instead be an outlier from the Indian source in which it has a 2nd PGM. Bead B08 – has a 1st PGM in the Yemen source and a 2nd in the Türkiye source. In this instance, there is not currently a second source from Yemen in the database that might strengthen the 1st PGM assignment. The 2nd PGM of Türkiye does, however, serve to indicate that the bead does not compositionally resemble agate from either Indian or northeast African sources. SI Fig. 8 is a bivariate plot of the first and second discriminant scores generated by this CDA. The 22 elements utilized in the CDA (listed on the plot itself) were selected at this and all stages using Wilks’ Lambda stepwise method. The six Banganarti beads are shown in relation to the 25 sources. Although in this two-dimensional representation there is a moderate amount of visual overlap between datapoints representing the geologic sources within the different regions under examination, all artifacts plot among the sources in which they had 1st PGMs.

When the 20 beads that were analyzed from Dongola are compared to the 25 agate sources using CDA, the resulting PGMs are quite variable (SI Appendix 4, Section A). Just over half (11 of 20) are assigned a 1st PGM in one of the Indian agate sources. Nine of those 11 also had a 2nd PGM in an Indian source. Of the remaining Dongola nine beads not assigned to India, four have a 1st PGM in the Yemen source, three in one of the Egyptian sources, one each in Sudan and Iran. SI Fig. 8 is a bivariate plot of this CDA. As with the Banganarti beads, most of those from Dongola fall among the groups of datapoints representing their 1st PGM assignments. There are exceptions like beads D26, D34, D94, and D136, which despite having 1st PGMs in the Yemen source plot distinctly apart from it on the figure, seemingly among datapoints belonging one of the Egyptian (Stele Ridge) or Turkish (Ankara) sources. The visually incongruent positioning of four beads is certainly due, in part, to limitations imposed by a two-dimensional representation of multi-dimensional data. However, it also indicates that even though out of the 25 sources to which they were compared at this stage these beads are most compositionally related to samples from Yemen, they are not chemically analogous to it. This could simply mean that all are compositional outliers that genuinely belong to the Yemen agate source. Alternately, they might they be from a geologically related occurrence that is also in the Yemen region or, perhaps, in similar terrain on the opposite side of the Red Sea Rift in northeast Africa. It is also possible that they are from a wholly unrelated source and/or region that is not currently represented in the dataset.

**Fig. 13:**
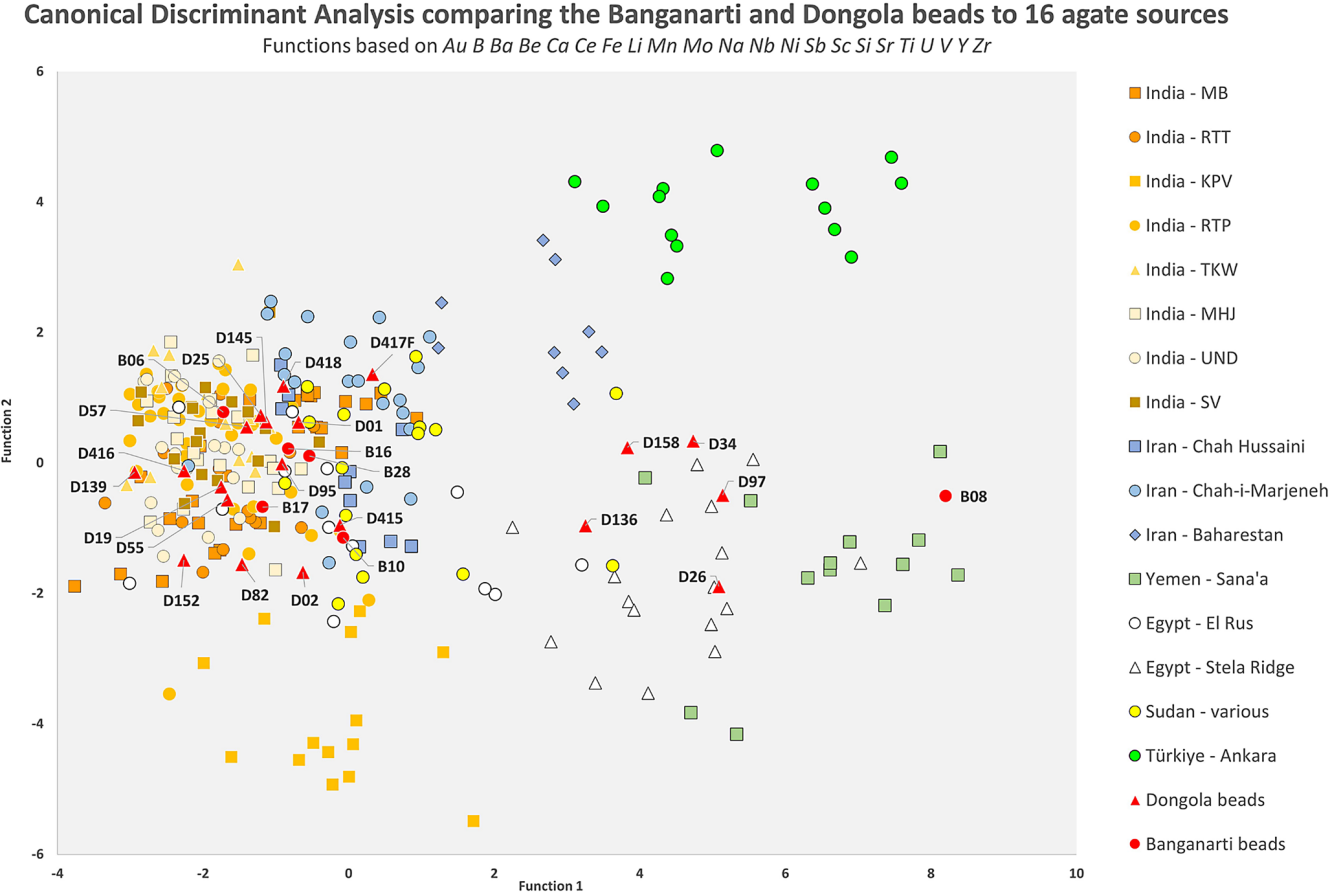
CDA Plot using 16 sources for beads from Banganarti and Dongola. Functions based on the 22 elements Au B Ba Be Ca Ce Fe Li Mn Mo Na Nb Ni Sb Sc Si Sr Ti UV Y Zr

For the next stage of analysis, those agate sources in the dataset that were not assigned to the Dongola and Banganarti beads as either a 1st or 2nd PGM were removed from consideration. This process left a total of 16 sources – eight sources in India, three in Iran, two in Egypt and one each in Sudan, Türkiye and Yemen. Paring down the dataset in this way allows us to focus on maximizing discrimination of the most geochemically pertinent sources and, possibly, revise the PGMs of beads that might be misassigned outliers. When a new CDA was undertaken comparing the six Banganarti beads to the 16 sources (Fig. 13), the PGMs the beads did indeed shift to a moderate degree (SI Appendix 4, Section B). Now, three beads are assigned to a source in India, and one each to sources in Yemen, Egypt and Iran. The bead assigned to Egypt – B17 – now has a 2nd PGM in Sudan, which serves to strengthen an interpretation that the raw material for that bead is likely from northeast Africa. When a new CDA was done comparing the twenty Dongola beads to the 16 sources (Fig. 13), some PGMs also changed (SI Appendix 4, Section B). Twelve beads now have a 1st PGM in a source in India, nine of which also has a 2nd PGM in that region. Of the remaining eight Dongola beads, two have 1st PGMs in a northeast African source (Egypt or Sudan), three in Yemen, two in Iran and one in Türkiye.

In the end, the patterns of carnelian bead acquisition at both Dongola and Banganarti appear to be broadly similar. Around half of the beads analyzed from each clearly seem to have been made of raw material coming from a source in India. Many of the remaining beads appear to be composed of carnelian most closely resembling agate-carnelian from sources (in Egypt, Sudan or Yemen) that straddle the Red Sea Rift zone of northeast Africa and southwest Arabia. Finally, a handful of beads were assigned to Iran and Türkiye. While these might genuinely be composed of raw materials from those regions, their assignments are somewhat tenuous. It is possible that these beads are misassigned outliers from sources in the Indian Subcontinent and/or Red Sea Rift Zone. However, it is also quite possible that they are from a source elsewhere in Africa that is not yet represented in the agate-carnelian database.

## Discussion and conclusion

This study of a small collection of carnelian beads from Sudan demonstrate the immense potential for better understanding the complex network of trade that brought carnelian beads to the upper Nile River Valley in Sudan from multiple regions, including South Asia, Iran, Türkiye, Yemen and Lower Egypt. Many of the imported carnelian beads probably arrived in Northeast Africa through the Red Sea ports of Suakin (founded in the 9th century) or Aidhab, in use as a port at least from the time of the Fatimid conquest of Egypt in CE 969 (Phillips 2019; Power 2008). While most of the carnelian beads appear to source to workshops in South Asia, and most likely the western coast of peninsular India, the production of carnelian beads in other regions, but using diamond drilling technology of South Asia needs to be further investigated.

The lack of historical documents that provide any information on the spread of diamond drilling technology from South Asia to other regions is surprising, since there are references to the trade in carnelian and agate products from India, starting from the Roman authors (Eichholz 1962) through to the Islamic travelers (Said 1989; Arkell 1936). The one question that needs to be resolved however, is that if diamond drilling did spread from South Asia to these other regions (i.e. Iran, Türkiye, Egypt, Yemen, etc.,) during the Achaemenid or later Islamic periods, why isn’t it still practiced in these regions? One would expect that some evidence for this highly specialized technology would be present in these regions historically and even today, but there are no published references to the use of double diamond drilling in any of the regions outside of South Asia. Single diamond drilling was documented in Afghanistan (Wright 1982) but not double diamond drilling. The only region outside of South Asia that has evidence for the use of double diamond drilling is in the agate processing and bead workshops of Idar-Oberstein, Germany. Although agate working to make ring pieces and inlay or other objects may date as early as the Roman period, the production of agate beads in Idar-Oberstein did not begin until the mid 1800s after the introduction of agate from Brazil (Kaspers 2016:15; Frazier et al. 1992). The double diamond drills used in Idar-Oberstein appear to have been copied from double diamond drills used in Khambhat and Nagara, Gujarat, India (on going study by Kenoyer). The double diamond drilling technology eventually was replaced in Idar-Oberstein with the introduction of electrical drilling technology.

The only way to further our knowledge of the spread of double diamond drilling would be to discover actual bead workshops in Yemen, lower Egypt, Türkiye or Iran that have the partly drilled beads and manufacturing waste from this type of bead production. The detailed analysis of manufacturing debris and comparisons with production debris in South Asian and Indian workshops would eventually help determine if the craftspeople were from South Asia itself or if the technology was adopted by local bead makers.

## Electronic supplementary material

Below is the link to the electronic supplementary material.

Supplementary Material 1

Supplementary Material 2

Supplementary Material 3

Supplementary Material 4

Supplementary Material 5

Supplementary Material 6

Supplementary Material 7

Supplementary Material 8

Supplementary Material 9

Supplementary Material 10

Supplementary Material 11

Supplementary Material 12

## Data Availability

The new data relevant to this article are presented in Supplementary Information Section.
