# Supplementary material for: Tracing the origins of Carnelian ornaments in Northeast Africa: morphological, technological and chemical compositional analyses of beads from medieval and post-medieval upper Nubia, Sudan
Source: Archaeol Anthropol Sci. 2025 May 8;17(6):121. doi: 10.1007/s12520-025-02228-0 (PMC12062086; doi:10.1007/s12520-025-02228-0)
Supplement: Supplementary file 4 — Supplementary Material 4 [file 12520_2025_2228_MOESM4_ESM.docx]

| **Supplemental Table 4. Predicted Group Memberships (PGMs) generated by Canonical Discriminant Analysis (CDA) comparing Banganarti and Dongola carnelian beads to agate sources in Africa and Eurasia** | | | | | | |
| --- | --- | --- | --- | --- | --- | --- |
|  | | **A**: 25 sources comparison | |  | **B**: 16 sources comparison | |
| **Site** | **Bead #** | **1st PGM** | **2nd PGM** |  | **1st PGM** | **2nd PGM** |
| Banganarti | B06 | India - MHJ | India - RTP |  | India - MHJ | India - RTP |
| Banganarti | B08 | Yemen | Turkey - Ankara |  | Yemen | Turkey - Ankara |
| Banganarti | B10 | India - MHJ | India - MB |  | India - MB | India - MHJ |
| Banganarti | B16 | India - TKW | India - MB |  | India - TKW | India - MB |
| Banganarti | B17 | Egypt - H1 | India - SV |  | Egypt - H1 | Egypt - ER |
| Banganarti | B28 | India - SV | India - MHJ |  | India - MB | India - MHJ |
| Dongola | D01 | India - SV | India - TKW |  | India - SV | India - TKW |
| Dongola | D02 | Egypt - ER | Egypt - H1 |  | Egypt - ER | Egypt - H1 |
| Dongola | D19 | India - MHJ | India - UND |  | India - MHJ | India - UND |
| Dongola | D25 | India - TKW | India - RTT |  | India - TKW | India - RTT |
| Dongola | D26 | India - KPV | Yemen |  | India - KPV | Yemen |
| Dongola | D34 | Yemen | Turkey - Ankara |  | Yemen | Turkey - Ankara |
| Dongola | D55 | Egypt - ER | Egypt - H1 |  | Egypt - ER | Egypt - H1 |
| Dongola | D57 | India - TKW | India - SV |  | India - TKW | India - RTT |
| Dongola | D82 | Iran - CM | Egypt - ER |  | Egypt - ER | Egypt - H1 |
| Dongola | D95 | Egypt - H2 | India - TKW |  | India - TKW | Egypt - H2 |
| Dongola | D97 | Yemen | Iran - BH |  | Yemen | Iran - BH |
| Dongola | D136 | Yemen | Egypt - SR |  | Yemen | Iran - BH |
| Dongola | D139 | India - MHJ | Egypt - H2 |  | India - MHJ | India - RTP |
| Dongola | D145 | Iran - CM | Sudan |  | Sudan | Iran - CM |
| Dongola | D152 | Egypt - ER | Sudan |  | Egypt - ER | Egypt - H1 |
| Dongola | D158 | Yemen | Egypt - SR |  | Yemen | Sudan |
| Dongola | D415 | India - MHJ | India - MB |  | India - MHJ | India - RTP |
| Dongola | D416 | India - TKW | India - MHJ |  | India - TKW | India - RTT |
| Dongola | D417 | Iran - CM | Sudan |  | India - MB | Iran - CM |
| Dongola | D418 | India - MB | India - RTT |  | India - MB | India - MHJ |
