# Supplementary figures and images for: Tracing the origins of Carnelian ornaments in Northeast Africa: morphological, technological and chemical compositional analyses of beads from medieval and post-medieval upper Nubia, Sudan
Source: Archaeol Anthropol Sci. 2025 May 8;17(6):121. doi: 10.1007/s12520-025-02228-0 (PMC12062086; doi:10.1007/s12520-025-02228-0)

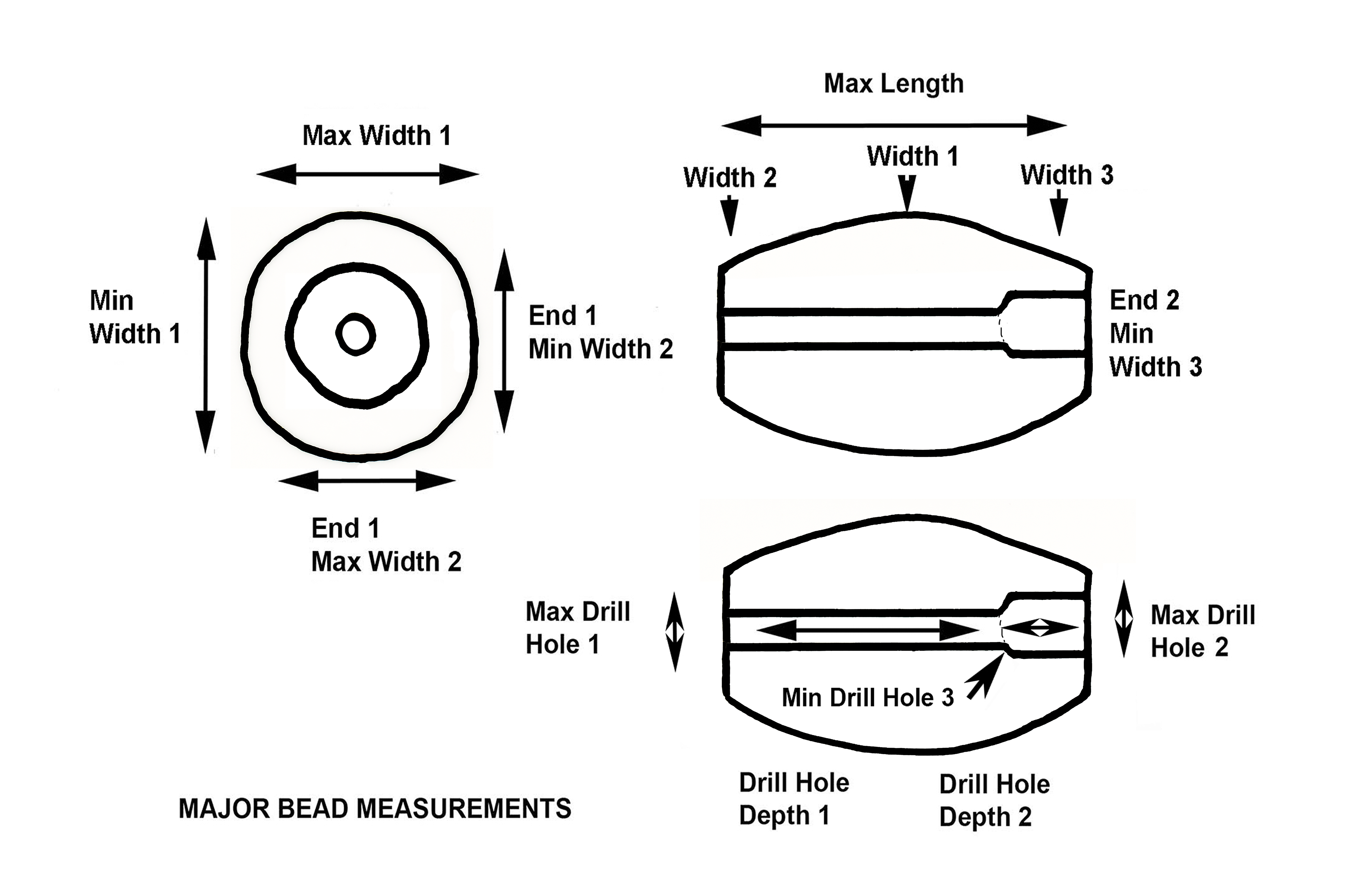

Supplement: Supplementary file 5 — Supplementary Material 5 [file 12520_2025_2228_MOESM5_ESM.tif]

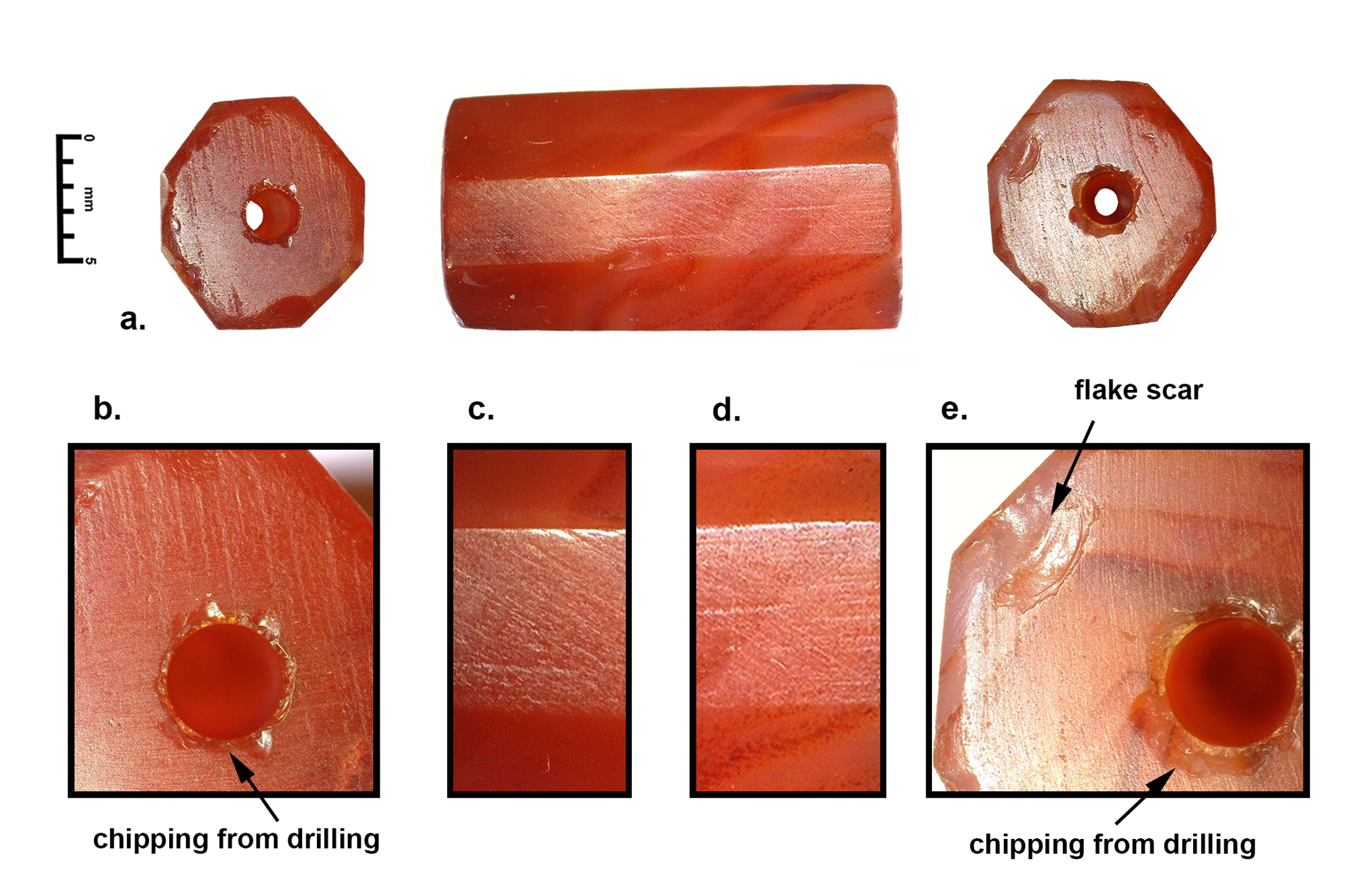

Supplement: Supplementary file 6 — Supplementary Material 6 [file 12520_2025_2228_MOESM6_ESM.tif]

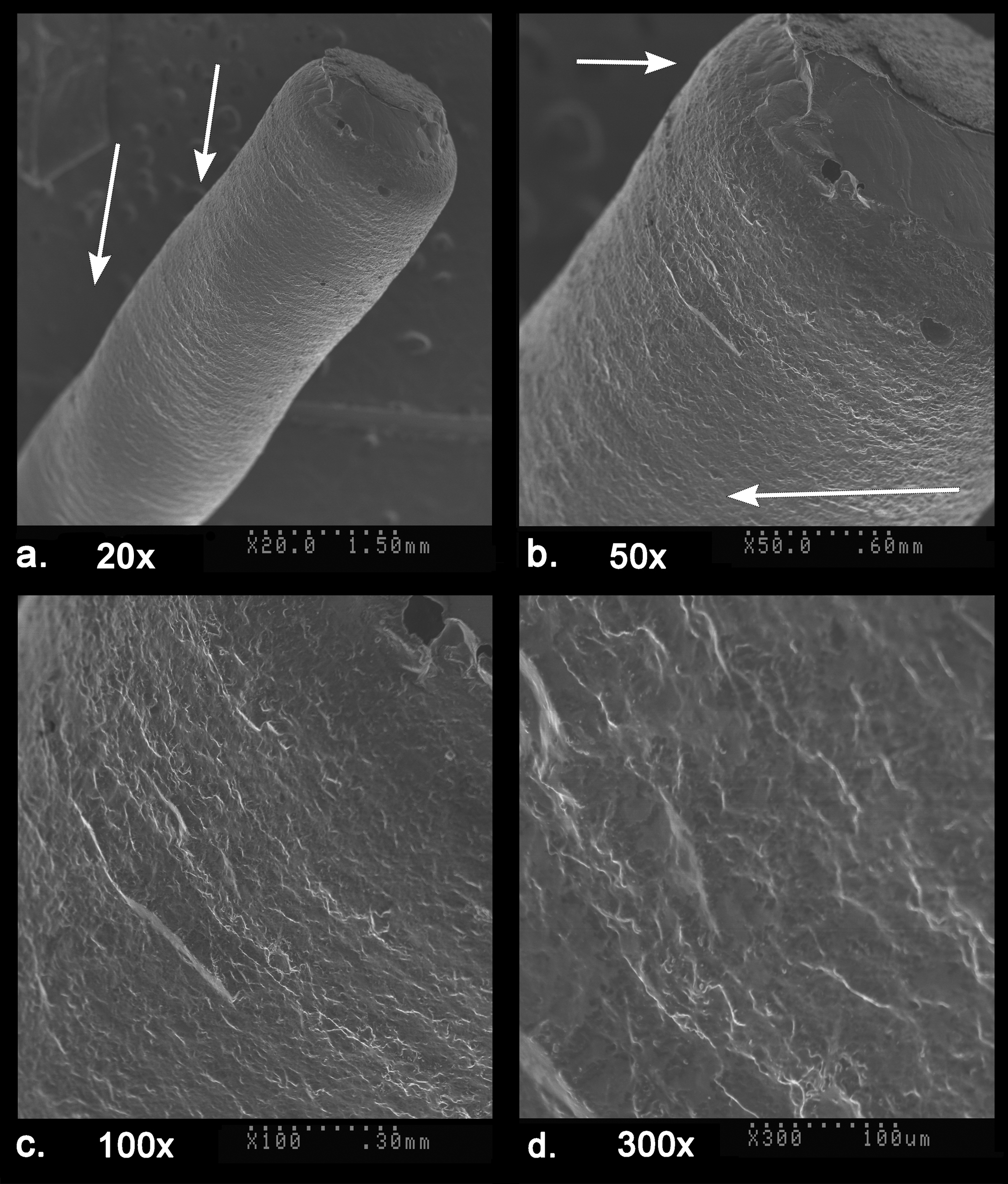

Supplement: Supplementary file 7 — Supplementary Material 7 [file 12520_2025_2228_MOESM7_ESM.tif]

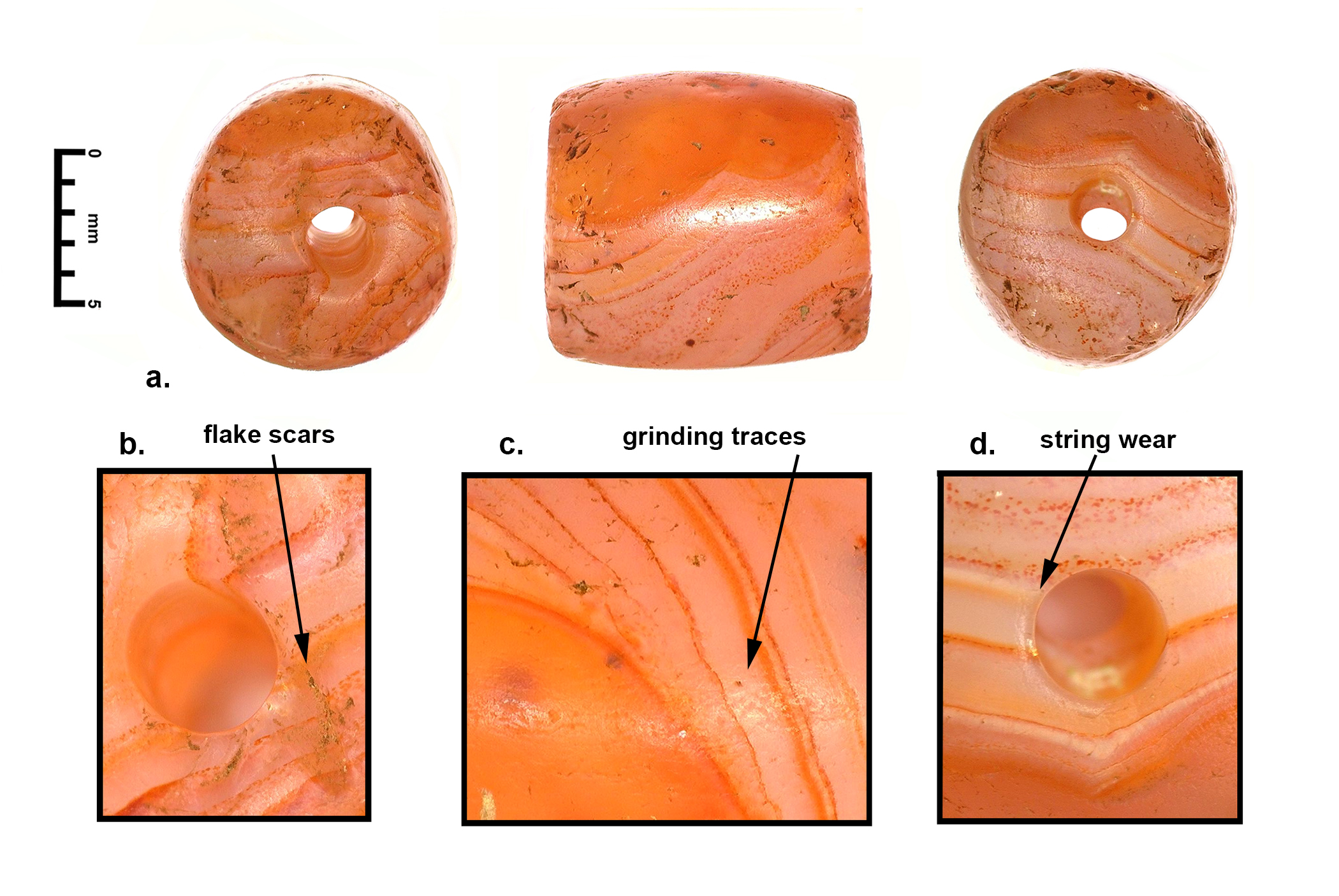

Supplement: Supplementary file 8 — Supplementary Material 8 [file 12520_2025_2228_MOESM8_ESM.tif]

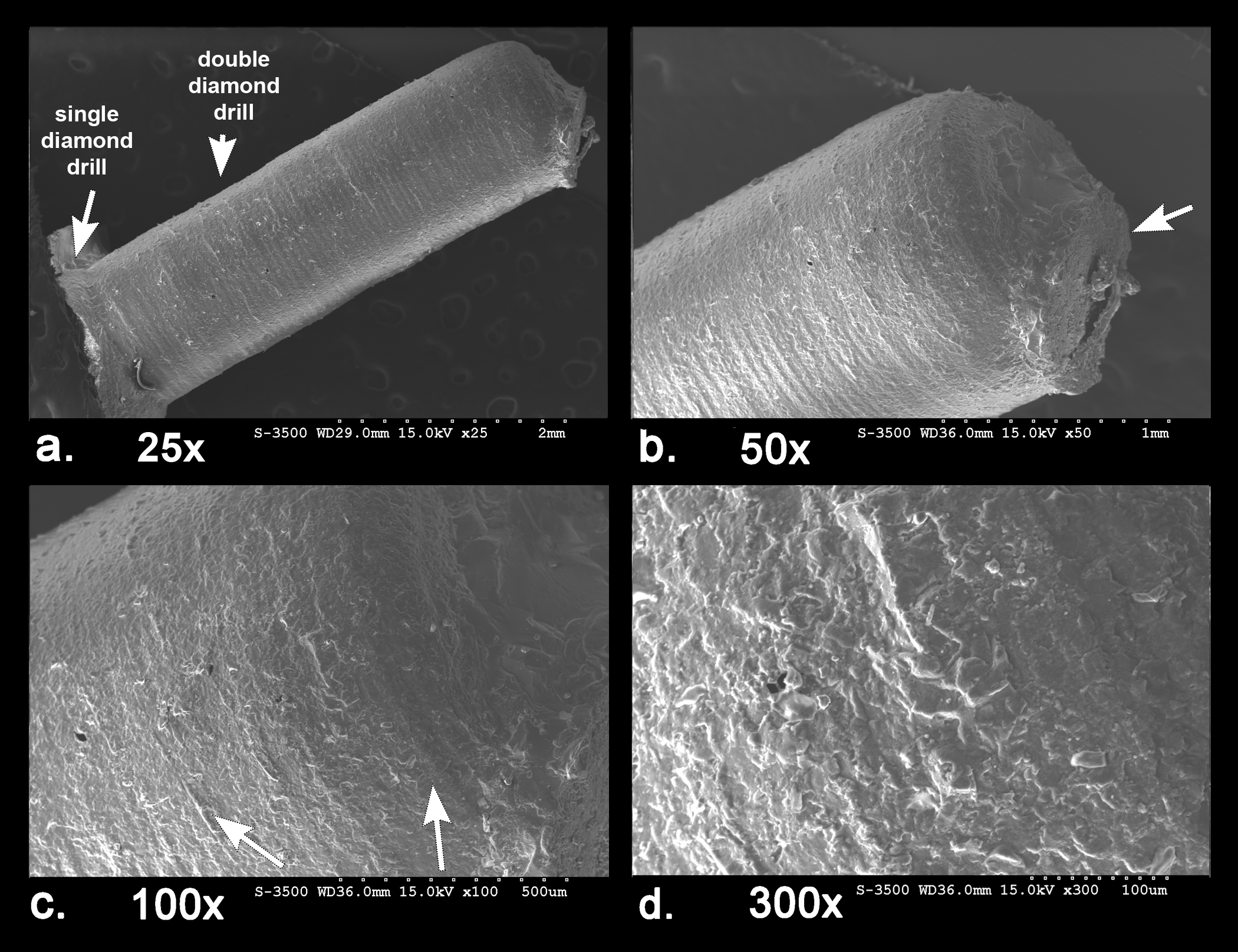

Supplement: Supplementary file 9 — Supplementary Material 9 [file 12520_2025_2228_MOESM9_ESM.tif]

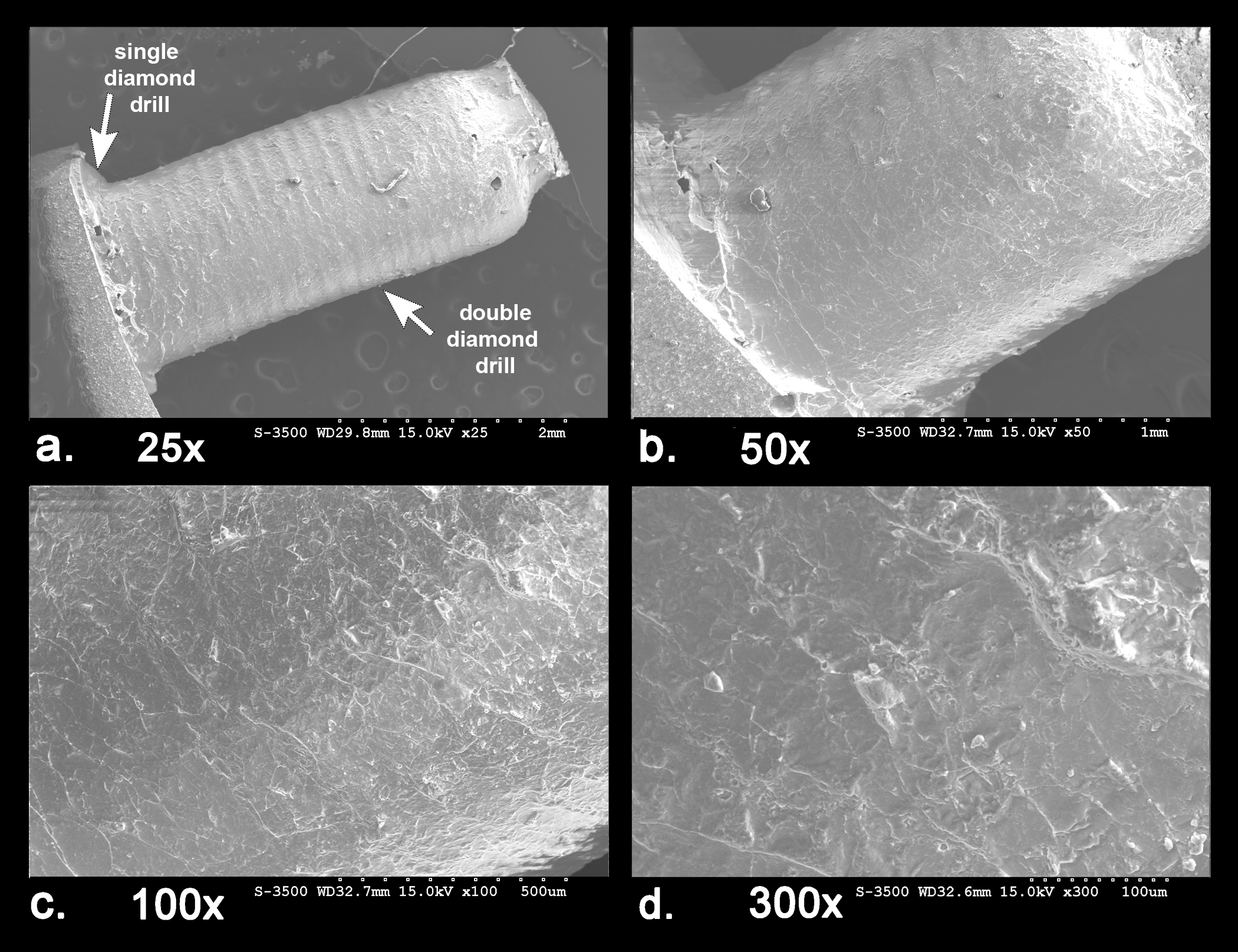

Supplement: Supplementary file 10 — Supplementary Material 10 [file 12520_2025_2228_MOESM10_ESM.tif]

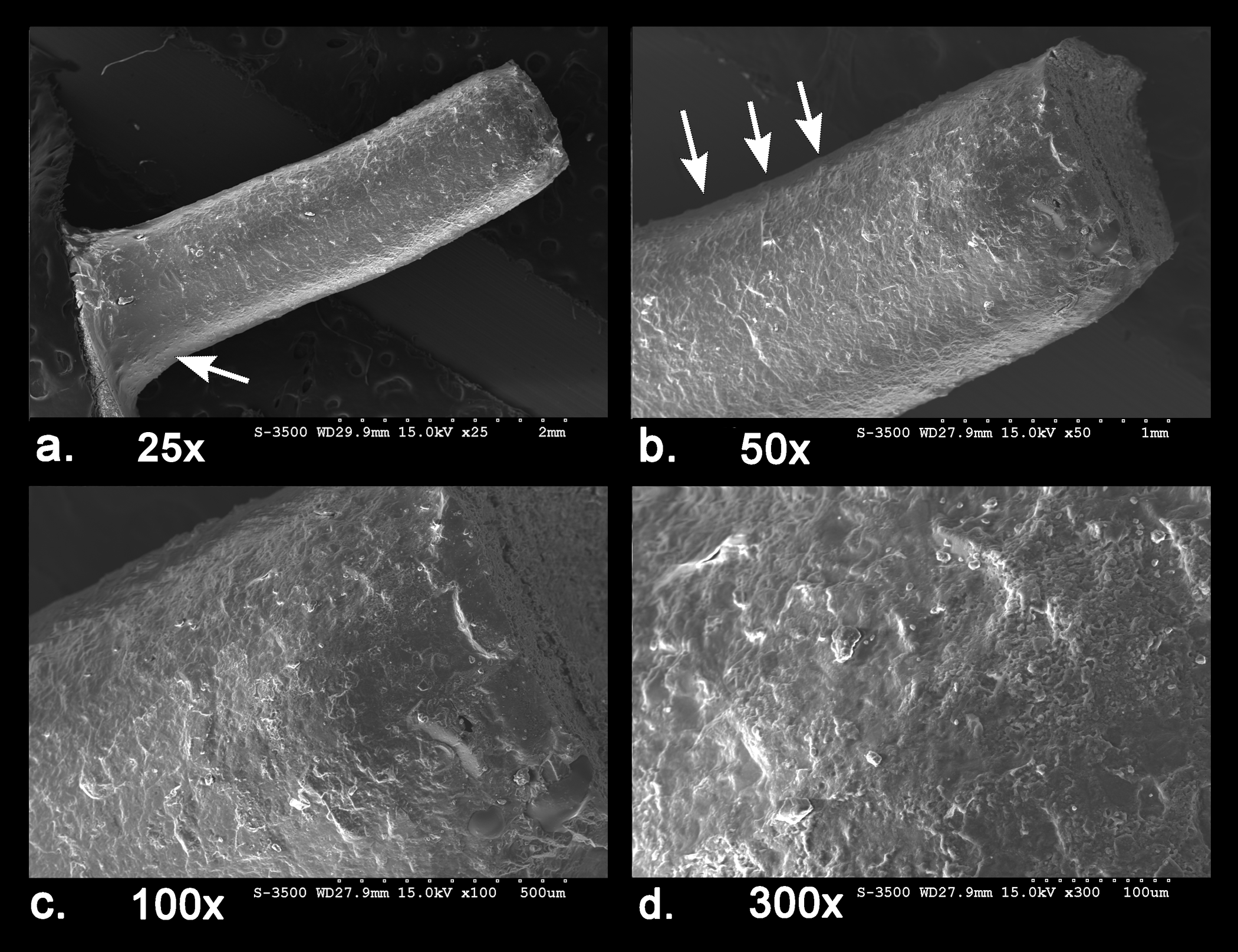

Supplement: Supplementary file 11 — Supplementary Material 11 [file 12520_2025_2228_MOESM11_ESM.tif]

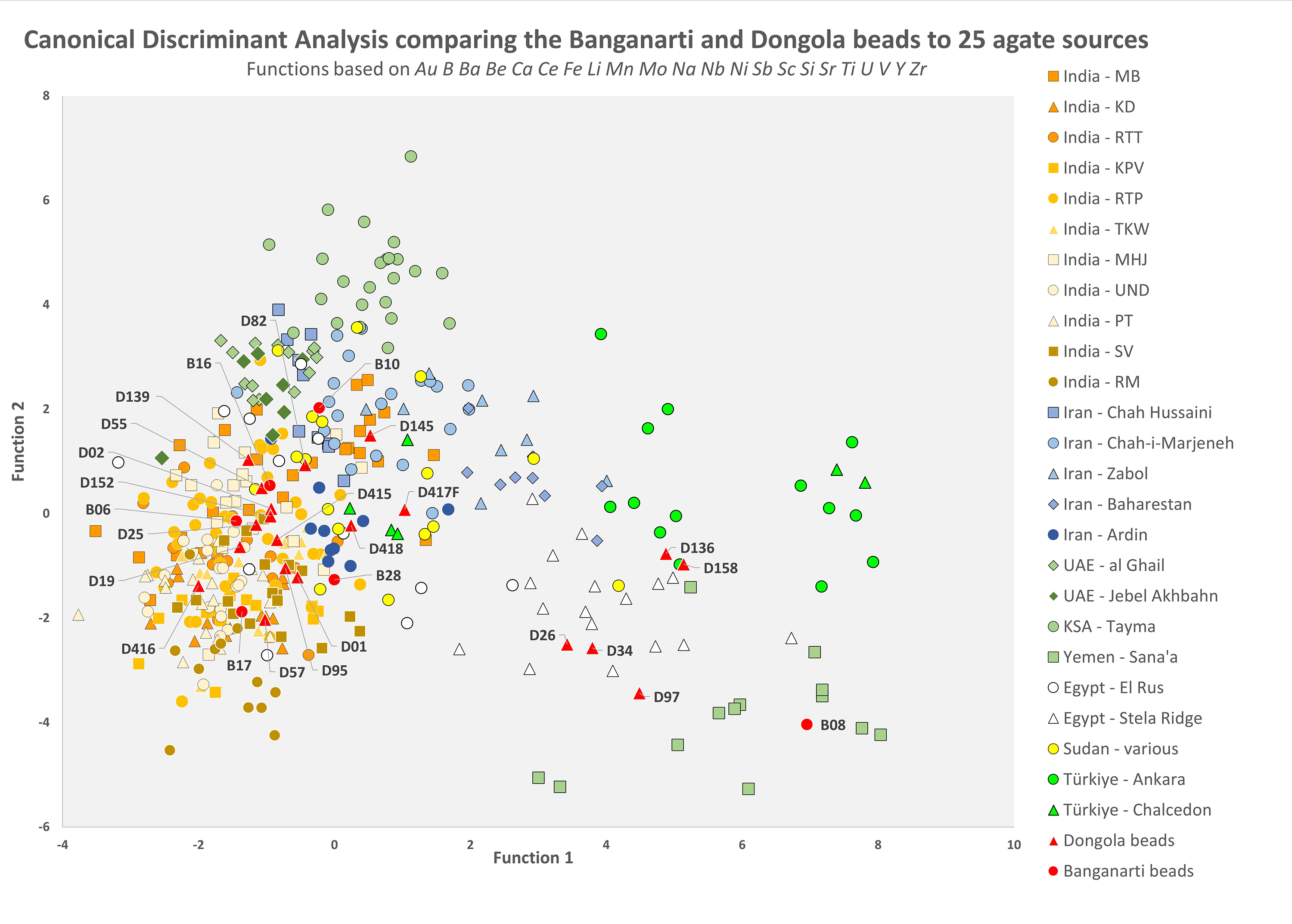

Supplement: Supplementary file 12 — Supplementary Material 12 [file 12520_2025_2228_MOESM12_ESM.tif]
